# Perfluorooctanoic acid (PFOA) induces lipid accumulation, oxidative stress, and reduced neurogenesis in primary human neuronal progenitor cells

**DOI:** 10.3389/ftox.2026.1814052

**Published:** 2026-05-15

**Authors:** William P. Marinello, Mark J. Zylka

**Affiliations:** 1 UNC Neuroscience Center, The University of North Carolina at Chapel Hill, Chapel Hill, NC, United States; 2 Department of Cell Biology and Physiology, The University of North Carolina at Chapel Hill, Chapel Hill, NC, United States; 3 Carolina Institute for Developmental Disabilities, The University of North Carolina at Chapel Hill, Chapel Hill, NC, United States

**Keywords:** brain, development, neuron, perfluoroalkyl, PFAS, PFOA, progenitor

## Abstract

**Introduction:**

Per- and polyfluoroalkyl substances (PFAS), particularly perfluorooctanoic acid (PFOA), are persistent environmental contaminants known for bioaccumulation and adverse health effects, including neurodevelopmental toxicity. This study investigated the impact of PFOA on primary human neuronal progenitor cells (phNPCs) derived from fetal brain tissue from genetically diverse donors, focusing on lipid metabolism and neuronal differentiation.

**Methods:**

phNPCs were exposed *in vitro* to PFOA at high concentrations (10,000–156 μM range) to determine cell viability and cytotoxicity using Alamar blue and lactate dehydrogenase (LDH) assays, respectively. Further experiments were conducted in 300–0.3 μM range where no effects on cell viability or cytotoxicity were observed. phNPCs were treated acutely (2 days) and assessed for changes in lipid droplet accumulation, fatty acid metabolism, lipid peroxidation, mitochondrial damage, and proliferation (EdU, Ki67, pHH3 staining). phNPCs were then exposed to PFOA for 14-days in neuronal differentiation media and assessed for changes in neuronal gene expression using quantitative reverse transcription polymerase chain reaction (RT-qPCR) and MAP2 protein expression and neuronal morphology using high content imaging. To assess differences in cytotoxicity between neuronal progenitors and neurons, fully differentiated neurons and phNPCs were both exposed to high concentrations (10,000–156 μM range) for 14 days and assessed for impacts on cell viability and death using Alamar Blue assays and flow cytometry using Calcein-AM/7-AAD stained cells.

**Results:**

Acute PFOA exposure induced dose-dependent lipid droplet accumulation, increased fatty acid uptake, reduced lipid turnover, elevated lipid peroxidation, mitochondrial reactive oxygen species, and fragmented mitochondrial morphology. The PFOA-induced lipid droplet accumulation was attenuated by inhibition of autophagy and lipolysis pathways, suggesting PFOA-induced lipotoxicity. PFOA exposure had minimal effects on phNPC proliferation but 14-day exposure during neuronal differentiation reduced MAP2-positive neurons, neuronal branching and gene expression of neuronal markers (*TUBB3, SYN1, MAP2*), while increasing the gene expression of progenitor-associated *FABP7*. Principal component analysis revealed PFOA-exposed cells exhibited intermediate gene expression between progenitors and mature neurons. Treatment of fully differentiated neurons during the same time window resulted in increased death cell and reduced viability compared phNPCs, suggesting neurons are more susceptible to PFOA cytotoxicity. Across donors, greater PFOA-induced lipid accumulation negatively correlated with neuronal differentiation outcomes.

**Discussion:**

These findings indicate that PFOA disrupts human neurodevelopment primarily by impairing neuronal differentiation, potentially through lipotoxicity and mitochondrial stress, highlighting a mechanistic link between dysregulated lipid metabolism and reduced neurogenesis.

## 1 Introduction

Per- and poly-fluoroalkyl substances (PFAS) are a diverse group of fluorinated compounds widely used in nonstick cookware, firefighting foams, personal care products, pesticides, and water-repellent clothing (Glüge et al., 2020). Known as “forever chemicals,” they resist degradation, bioaccumulate, and persist due to stable carbon-fluorine bonds and amphiphilic properties (Evich et al., 2022; Panieri et al., 2022). PFAS are detected in human blood, milk, urine, hair, and umbilical cord blood worldwide (Papadopoulou et al., 2016; Mamsen et al., 2017; Ruan et al., 2019; Kotlarz et al., 2020; Li et al., 2021; Domingo, 2025), with long half-lives (e.g., 5–8 years for some) due to poor metabolism (Rosato et al., 2024). Long-chain PFAS like perfluorooctanoic acid (PFOA) pose significant health risks, including hepatotoxicity, lipid dysregulation, immunosuppression, developmental issues, and cancer (Barry et al., 2013; Consonni et al., 2013; Johnson et al., 2014; Li et al., 2021; Zhang et al., 2023)). Despite voluntary phase-outs in the early 2000s, PFOA persists in humans, with longer half-lives than short-chain alternatives (Calafat et al., 2019).

An area of particular concern is neurodevelopment. Longer-chain PFAS like PFOA cross the blood-brain barrier (Dassuncao et al., 2019; Yu et al., 2020) and accumulate in brain regions (Greaves et al., 2013; Eggers Pedersen et al., 2015). Epidemiological links to neurodevelopmental disorders are mixed (Gao et al., 2023; Yao et al., 2023; Ames et al., 2025), but PFAS disrupt fetal weight, thyroid hormones, and placental function—critical for brain development (Liew et al., 2018; Blake and Fenton, 2020; Du et al., 2024). In rodents, gestational or postnatal PFOA exposure causes hyperactivity, reduced habituation/motor function, altered neurotransmitters (norepinephrine, dopamine, glutamate), disrupted calcium homeostasis, causes oxidative stress, and neuronal apoptosis (Johansson et al., 2008; Cheng et al., 2013; Sobolewski et al., 2014; Goulding et al., 2017; Brown-Leung and Cannon, 2022; Starnes et al., 2022).

Recent studies show PFOA disrupts neuronal differentiation. PFOA alters dopaminergic neuron development in human induced pluripotent stem cells (iPSCs) (Di Nisio et al., 2022), nuclear morphology, neuronal networks, and calcium activity in hiPSC-derived neurons (Wu et al., 2024), and neuronal marker expression in mouse embryonic stem cells (Yin et al., 2018). Our lab recently demonstrated that gestational PFOA exposure altered neuronal progenitor cell (NPC) proliferation and neuronal differentiation by altering the cell cycle in NPCs of male and female mice (Hu et al., 2025).

Mechanistically, PFOA binds the peroxisome proliferators-activated receptor (PPAR) receptors and fatty acid-binding proteins (Vanden Heuvel et al., 2006; Takacs and Abbott, 2007; Wolf et al., 2014; Zhao et al., 2023). These targets are important for fatty acid transport, lipid metabolism, beta oxidation and mitochondrial function. Lipid droplets (LDs) are essential for NPC-to-neuron differentiation (Knobloch et al., 2017; Ramosaj et al., 2021; Petrelli et al., 2023), suggesting a link between the disruption of lipid metabolism and neuronal differentiation. Here, we used genetically diverse primary human NPCs (phNPCs) to better understand how PFOA affects neuronal differentiation, NPC proliferation, cell-type proportions and toxicity during differentiation, sex-specific effects, and alterations in lipid metabolism.

## 2 Materials and methods

### 2.1 Chemicals

PFAS chemicals PFBS (Cas. 375-73-5, Sigma, 562629), PFBA (Cas. 375-22-4, Sigma, 164194), PFHxA (Cas. 307-24-4, TCI, U0067), PFOS (Cas. 1763-23-1, Sigma, 33607), and PFOA (Cas. 335-67-1, TCI, P0764) were dissolved in MeOH and K^+^PFHxS (Cas. 3871-99-6, TRC, T774700) was dissolved in DMSO at a concentration of 100 mM and stored at −20 °C for preservation.

Ferroptosis-inhibitor Ferrostatin-1 (Fer1, MedChemExpress, HY-100579, conc: 10 mM), necrosis-inhibitor Necrostatin-1 (Nec1, MedChemExpress, HY-15760, conc: 10 mM), apoptosis-inhibitor Q-VD-OPh (7VD, MedChemExpress, HY-12305, conc: 10 mM), insecticide rotenone (Rot, Sigma, 45656, conc: 10 mM), adipose triglyceride lipase inhibitor atglistatin (ATGL, Sigma, SML1075, concentration: 20 mM), fatty acid oxidation inhibitor etomoxir (Eto, MedChemExpress, HY-50202A, concentration: 20 mM), diacylglycerol acyltransferase (DGAT)-1 inhibitor T863 (DGAT1i, Sigma, SML0539, concentration: 10 mM), DGAT-2 inhibitor PF-06427878 (DGAT2i, Sigma, PZ0412, concentration: 10 mM), and autophagy inhibitor 3-Methyladenine (MedChemExpress, HY-19312, concentration: 20 mM) were dissolved in DMSO while fatty acid synthesis inhibitor cerulenin (FASNi, Sigma, C2389, concentration: 22 mM) was dissolved in MeOH and all were stored at −20 °C until further use. Lipid peroxidation positive control cumene hydroperoxide (CHP, Sigma, 247502) was dissolved in 100% EtOH at a concentration of 100 mM and stored at −80 °C. Oleic acid (OA) was prepared according to (Ramosaj et al., 2021) in ddH_2_O with 10% BSA (Sigma, A8806) and 50 mM NaOH at a final concentration of 10 mM. OA and vehicle (10% BSA) aliquots were stored at −20 °C.

### 2.2 Cell culture

We cultured and differentiated phNPCs as previously described (Stein et al., 2014; Wolter et al., 2020; Aygün et al., 2021; Liang et al., 2021). Biological sex was determined as described previously (Aygün et al., 2021) by SNP genotyping based on heterozygosity on the X chromosome and XIST expression. Briefly, low passage number cells were thawed and plated on Poly-L-ornithine- (PLO, Sigma, P3655)/Laminin-coated (LAM, Life Technologies, 23017015) 6-well plates with NPC media composed of Neurobasal A (Life Technologies; 10888–022) with 100 μg/mL Primocin (Invivogen; ant-pm-2), 10% BIT 9500 (Stemcell Technologies; 09500), 1% Glutamax (100×) (Life Technologies; 35050061), 1 μg/mL Heparin (Sigma-Aldrich; H3393–10KU), 20 μg/mL EGF/FGF (Life Technologies; PHG0313/PHG0023), 2 ng/mL LIF (Life Technologies; PHC9481) and 20 ng/mL PDGF (Life Technologies; PHG1034) at 37 °C with 5% CO_2_. After thawing, cells were passaged at least twice before splitting for experiments.

For neuronal differentiation, phNPCs were split onto PLO-/LAM-coated plates into NPC media. Then, 24 h post-seeding, media was switched to differentiation media containing Neurobasal A, 100 μg/mL Primocin, 2% B27 (Life Technologies, 17504-044), 1% Glutamax (100×), 10 ng/mL NT-3 (Life Technologies; PHC7036) and 10 ng/mL BDNF (Life Technologies; PHC7074). Fresh media was exchanged every 2 days.

### 2.3 Chemical exposure

In all experiments, phNPCs were plated and after 24 h, exposed to the indicated chemical. In initial acute exposure experiments, PFAS chemicals was prepared in cell culture media by creating a 10 mM solution followed by 1:2 dilutions (10,000–156 μM range). The phNPCs were exposed to PFOA or vehicle (MeOH) for 48 h and then assessed for impacts to cell viability using Alamar Blue (Thermo-Fisher, A50100) and lactate dehydrogenase (LDH) assays (Thermo-Fisher, 88953) to generate a dose-response curve determine EC_50_ viability for select PFAS compounds in this specific cell type. All further experiments except MitoSox and lipid peroxidation were conducted at concentrations where no changes in viability were observed.

PFOA was prepared in the cell culture media by creating 300 μM and 100 μM (or 3,000 μM and 1,000 μM in MitoSOX and Lipid peroxidation experiments) solutions followed by 1:10 dilutions in media. This concentration range was chosen to be consistent with a previous *in vitro* PFAS neurotoxicity screening study done at the US EPA (Carstens et al., 2023). The final concentration of vehicle control was the amount used at the highest concentration which was 0.15% (v/v) in most experiments.

In acute exposure experiments, phNPCs were exposed to PFOA or vehicle (MeOH) for 48 h and then assessed for lipid accumulation and oxidative stress and mitochondrial function using fluorescence microscopy. Additional acute experiments that examined how 48 h PFOA exposure impacted the lipid turnover and fatty acid transport are described in Section 5 Pulse-chase Experiments. For experiments longer than 48 h, cells were exposed to PFOA or vehicle with each new media change.

For experiments involving lipid metabolism inhibitors, cells were co-treated with the following lipid metabolism inhibitors or vehicle controls: 10 μM lipase inhibitor Atglistatin (ATGL), 10 μM fatty acid oxidation inhibitor Etomoxir (ETO), 1 μg/mL fatty acid synthase inhibitor cerulenin, 10 μM 3-autophagy inhibitor Methyladenine (3-MA), and 5 μM Diacylglycerol acyltransferase-1 (DGAT-1) inhibitor T863 and 5 μM DGAT-2 inhibitor PF-06424439. Doses of inhibitors were chosen based on previous studies demonstrating an impact on lipid metabolism (Singh et al., 2009; Brose et al., 2016; Nguyen et al., 2017; Ioannou et al., 2019; Ramosaj et al., 2021) and had no significant difference with vehicle controls in viability after 48 h exposure measured by Alamar blue assay.

### 2.4 Cell viability and cytotoxicity

Cells were seeded at 20,000 cells per well in a 96-well plate. For cell viability experiments using cell death inhibitors Fer1, Nec1, 7VD or vehicle (DMSO) was added to the media containing vehicle (CTRL) or PFOA at a final concentration of 10 μM. Cell viability and cytotoxicity were calculated using Alamar blue and LDH assays, respectively. For Alamar blue assay, after chemical treatment, the media was removed and replaced with Neurobasal A with 10% Alamar blue. This plate was incubated at 37 °C and 5% CO_2_ in a humidified atmosphere and fluorescent measurements read 4 h later. Alamar blue fluorescence (ABF) was quantified at the respective excitation and emission wavelength of 530 and 590 nm by a microplate reader (BMG LabTech, ClarioStar Plus). Each plate was run with positive control of 100% reduced form of Alamar Blue and a negative control of 0% reduced form of Alamar Blue.

For LDH assay, after chemical treatment 50 μL of cell culture media was transferred to a new 96-well plate and 50 μL of the reaction mixture was added, incubated at room temperature for 30 min protected from light then 50 μL stop solution was added and mixed by gentle tapping. Absorbance was measured at 490 nm and 680 nm and LDH activity was calculated as 490 nm value minus 680 nm. Each experiment was run with negative control that utilized sterile water instead of cell culture media and positive control that included cells lysed by the kit provided lysis buffer. The percent viability (Alamar Blue) and cytotoxicity (LDH assay) was calculated with the test value (either fluorescence or LDH activity) and appropriate controls as the percentage of compound test value minus the average test value of the negative control, divided by average test value of the positive control minus the average test value of the negative control. Experiments in each assay were performed in triplicate wells with each replicate performed on a different donor line.

### 2.5 Pulse-chase experiments

For the PFOA-pulse-chase experiments, 60,000 cells were seeded in 4 MatTek 35 mm glass bottom plates (MatTek, P35G-1.5-20-C), 24 h post-seeding, one plate was treated with vehicle CTRL and the three other plates were treated with 300 μM PFOA for 48 h. Post-treatment, the cells were washed once with PBS. One CTRL and one PFOA plate (0 h chase) were fixed, washed 2 times with PBS and stored at 4 °C. The media in the other two plates were replaced with NPC media and incubated for 24 or 48 h chase and then fixed and washed at the end of the timepoint and stained for lipid accumulation.

For the OA-pulse-chase experiments, 60,000 cells were seeded in 6 MatTek plates. Twenty-four hours after seeding, plates were treated with vehicle (CTRL) or 300 μM PFOA for 48 h. After treatment, the one plate of CTRL- and PFOA-exposed cells were fixed, washed 2 times with PBS and stored at 4 °C. The other plates were treated with 0.5 mM oleic acid (OA). After 5 h of OA treatment, the remaining plates were washed and either fixed (pulse) or replaced with NPC media for 24 h then fixed and stained for BODIPY and DAPI.

For the FA-pulse-chase experiments, 60,000 cells were seeded in 6 MatTek plates. Twenty-four hours after seeding, plates were treated with vehicle (CTRL) or 300 μM PFOA for 48 h. After treatment, the one plate of CTRL- and PFOA-exposed cells were fixed, washed two times with PBS and stored at 4 °C. The other plates were treated with 1 μM BODIPY C12 558/568 (Thermo-Fisher, D3835). After 16 h of BODIPY C12 treatment, the remaining plates were washed and either fixed at 6 h or 24 h post-washing and counterstained with DAPI.

### 2.6 Proliferation and quiescence

Cells were plated at 60,000 cells/well in 24-well plates for flow cytometry and 20,000 cells/well in 96-well plates for high content imaging. On the last day of PFOA exposure, cells were treated with 10 μM EdU with a 10% media addition and incubated for 2 h. For flow cytometry, the cells were lifted off the plate using Accutase (Sigma, A6964) and placed into polypropylene round-bottom tubes. The cells were fixed with 4% PFA for 15 min at room temperature. EdU labeling was performed using the Click-iT EdU Cell Proliferation Kit (Thermo-Fisher, C10337) per manufacturer’s protocol. Total DNA content was labeled with the FxCycle Far Red dye (Thermo-Fisher, F10347). Cell suspensions were quantified using the Attune NxT 96-well Flow Cytometer (Thermo Fisher Scientific). For high content imaging assays, cells were fixed and labelled with EdU (as above) then washed and labelled using antibodies for Ki67 and phospho-histone H3 (pHH3) and imaged on the high content imager.

Quiescence was induced as previously described (Knobloch et al., 2017; Ramosaj et al., 2021). Cells were plated at 20,000 cells/well in 96-well plates for high content imaging in NPC media. After 24 h, media was changed to either fresh NPC media or quiescence (qNPC) media which had the same composition as NPC media except the removal of EGF and addition of BMP4 (50 ng/mL). After 48 h, wells that previously contained NPC media were either changed with fresh NPC media (NPC condition) or quiescence media (qNPC entry condition) and wells that previously contained qNPC media were maintained in qNPC media (qNPC condition) or switched to NPC media to exit quiescence (qNPC exit condition). Co-exposure to PFOA occurred during this 96 h time frame with each media change, after which cells were fixed and stained with antibodies for Ki67 and pHH3 and prepared for high-content imaging.

### 2.7 Neuronal differentiation

For neuronal differentiation, cells were plated at 90,000 cells/well in 12-well plates for RNA extraction and 20,000 cells/well in 96-well plates for high content imaging. Twenty-four hours after plating, the media was changed to neuronal differentiation media with PFOA or vehicle with media changes every 2 days for 14 days. At the end of the exposure window, cells were harvested for RNA extraction or fixed and stained for high content imaging. As a negative control for neuronal differentiation, cells grown in NPC media were collected for RNA or fixed for imaging.

To assess cell-type differences in viability/cytotoxicity between neurons and NPCs, experiments used either phNPCs after splitting or 5-week differentiated neurons. First, phNPCs were plated in either 60,000 cells/well in 24-well plates for flow cytometry or 20,000 cells/well in 96-well plates for Alamar blue assay. For NPCs, cells were exposed 1 day after plating for 14 days in NPC media. For neurons, phNPCs were cultured in Neuronal Differentiation Media for 5 weeks after which exposure to PFOA began and continued for 14 days. For Alamar blue plates, media was changed every 2 days and replaced with PFOA containing media. For plates for flow cytometry, since dead cells detached from the plates after PFOA exposure, media was collected at Day 4, Day 7, Day 10 and Day 14 to quantify floating dead cells and replaced with PFOA- or vehicle-containing media. On Day 14, cells were lifted using Accutase. On each collection day, cells were placed into polypropylene round-bottom tubes and immediately stained with 7-AAD (Thermo-Fisher, 00-6993-50) and Calcein-AM (Thermo-Fisher, 65-0853-39). Cells were resuspended in 1 mL PBS + 1% BSA and incubated with 1 μM Calcein-AM for 30 min at room temperature, followed by the addition of 5 μL 7-AAD (final concentration 10 μM) to the PBS + 1% BSA solution for 15 min. Cells were then analyzed on the Attune NxT 96-well Flow Cytometer.

### 2.8 Microscopy

Microscopy was performed using either high content imaging or confocal microscopy. High content imaging experiments were performed in PLO/LAM-coated 96-well polystyrene plates (Griener 655090) with images acquired on the GE IN Cell Analyzer, an automated fluorescence microscope with sCMOS camera. Images were acquired at ×20 magnification with 10–12 regions of interest (ROI) imaged per well. For each high content imaging plate, conditions were run on duplicate wells, and each replicate was performed on a different donor line.

Confocal microscopy was used for experiments requiring higher resolution to image cellular structures such as mitochondria and LDs. These experiments were performed on either Ibidi chamber slides (Ibidi, 81816) or MatTek glass bottom chamber wells (MatTek, P35G-1.5-20-C) and acquired on the Zeiss 880 confocal microscope at ×40 magnification with ×2 zoom. For these experiments, five to eight ROIs were imaged per well and each cell was treated as a replicate with each experiment being performed on at least three donor cell lines.

#### 2.8.1 Immunostaining

Cells were washed once with PBS then fixed with 4% PFA (37 °C) for 15–30 min at RT, washed two times with PBS for 10 min each and stored at 4 °C. For immunolabeling, cells were rinsed in PBS and incubated in blocking solution (5% normal donkey serum, 0.1% Triton-X100 in PBS) for 1 h at room temperature. Primary antibodies were diluted in blocking solution and incubated overnight at 4 °C. The following antibodies and conditions were utilized for immunolabelling: rabbit anti-SOX2 (Sigma-Aldrich, AB5603; 1:1,000), mouse anti-MAP2 (Sigma-Aldrich, MAB3418; 1:1,000) mouse anti-Ki-67 (BD Biosceinces, 550609, 1:500), and rabbit anti-pHH3 (Cell Signaling, 9701, 1:500). The cells were rinsed three times with PBS with 0.1% Triton-X100 (PBS/T) for 10 min per wash and incubated with secondary antibodies in blocking solution for 1 h at room temperature. Secondary antibodies included: donkey anti-mouse IgG Alexa Fluor 488 (ThermoFisher Scientific, A21202; 1:1,000), donkey anti-rabbit IgG Alexa Fluor 568 (ThermoFisher Scientific, A10042; 1:1,000), donkey anti-mouse IgG Alexa Fluor 568 (ThermoFisher Scientific, A10037; 1:1,000), and donkey anti-rabbit IgG Alexa Fluor 647 (ThermoFisher Scientific, A31573; 1:1,000). The cells were then rinsed with PBS/T three times for 10 min each and counterstained with 1 μg/mL DAPI (Thermo-Fisher, 62248) in PBS.

#### 2.8.2 Lipid droplet accumulation

Cells were plated in 20,000 cells/well in ibidi plates or 60,000 cells/well in MatTek plates and treated with 30, 100, or 300 μM PFOA for 48 h. Cells were fixed with 4% PFA (37 °C) for 15–30 min at RT, washed two times with PBS and LDs were stained for 1 h with 1 μg/mL of BODIPY 493/503 (Thermo-Fisher, D3922) in PBS. The cells were then rinsed twice with PBS and counterstained for 30 min with 1 μg/mL DAPI solution in PBS.

#### 2.8.3 Lipid peroxidation measurement

NPCs were seeded at 20,000 cells/well in a 96-well plate and treated with vehicle, 30–3,000 μM PFOA, or 50 μM cumene hydroxide (CHP) as a positive control for 4 h then 5 μM BODIPY C11 581/591 (Thermo-Fisher, D3861) was added to the media for 30 min. After loading, cells were washed once with PBS and imaged in PBS with 5 μg/mL of Hoechst 33342 (Thermo-Fisher, H1399) at 37 °C and immediately imaged on the high content imager. Fluorescence imaging was performed using Texas Red (581/591 nm) and FITC (488/510 nm) filters.

#### 2.8.4 Mitochondrial ROS measurement

MitoSOX staining was performed for the detection of mitochondrial superoxide. NPCs were seeded at 20,000 cells/well in Ibidi plates and exposed to vehicle, 30–1,000 μM PFOA or 10 μM rotenone (Rot) as a positive control for 48 h. After treatment, NPC media was replaced with media containing 100 nM MitoView (Biotium, 70054) and 1 μM MitoSox (Thermo-Fisher, M36008) for 30 min at 37 °C. Cells were washed twice with HBSS and replaced with 5 μg/mL Hoechst 33342 in HBSS at 37 °C and immediately imaged on the confocal microscope.

#### 2.8.5 Fatty acid analog BODIPYC12 measurement

NPCs (60,000/well) were seeded in 6 MatTek plates (Described in 5.1 Pulse-chase experiments), after BODIPY C12 treatment, cells were washed twice with PBS and replaced with 5 μg/mL Hoechst 33342 in HBSS at 37 °C and immediately imaged on the confocal microscope.

#### 2.8.6 Image analysis

All images were analyzed in a semi-automated manner using CellProfiler (Version 4.2.1) with pipelines adapted from (Tian et al., 2019; Lickfett et al., 2022; Sokolov et al., 2023). Images were corrected for background signal. Minimum cross-entropy thresholding of the DAPI channel was used to identify nuclei of cells as primary objects. To determine if a nucleus was positive for a cell marker (ex. MAP2, SOX2, *etc.*), thresholding of the respective channel was used to create a binary image mask and nuclei that overlapped at least 40% with the binary mask were classified as positive cells. For analysis of neuronal morphology, neurites were suppressed, then using the propagation algorithm in CellProfiler, the cell body was identified using the nucleus as seed. The propagation algorithm works to identify cell boundaries by starting at the nucleus and moving until it touches another cell of the intensity is below the threshold. After cell bodies were delineated, images were then re-enhanced to identify axon projections. The image was then skeletonized to calculate total branch length, number of endpoints, and number of branches (trunk and non-trunk) for each neuron. For analysis of LDs with confocal images of phNPCs, like neurons, the nuclei were used as seeds then the propagation algorithm was of the BODIPY channel was used identify cell boundaries. Then, within cells minimum cross-entropy thresholding was used to define the LDs. For analysis of mitochondria morphology, images were imported into Fiji and each cell was analyzed using the Mitochondrial Network Analysis (MiNA) plugin using the default settings.

### 2.9 RNA extraction and RT-qPCR

Cells were washed with PBS and collected in TRIzol (Invitrogen, no. 15596026) for total RNA extraction. RNA was treated with ezDNase and reverse transcribed into cDNA using SuperScript IV VILO (Invitrogen, no. 11766050). All qPCR experiments were performed using SsoAdvanced Universal SYBR Green Supermix (Bio-Rad, no. 1725271) on a QuantStudio5 (Applied Biosystems). The following primers used were: *ACTB*-F: TCC​GCA​AAG​ACC​TGT​ACG​CC, *ACTB*-R: AGG​GGC​CGG​ACT​CGT​CAT​AC, *DCX*-F: TCA​GGG​AGT​GCG​TTA​CAT​TTA​C, *DCX*-R: GTT​GGG​ATT​GAC​ATT​CTT​GGT​G, *EIF4A2*-F: CGG​GAT​TGA​TGT​GCA​ACA​AGT​G, *EIF4A2*-R: ATG​GGC​ATC​TCC​TCC​ACT​GTA​G, *FABP7*-F: CCA​GCT​GGG​AGA​AGA​GTT​TG, *FABP7*-R: CTT​TGC​CAT​CCC​ATT​TCT​GT, *GAPDH*-F: GCT​TAG​CAC​CCC​TGG​CCA​AGG, *GAPDH*-R: CTT​GGC​AGC​GCC​AGT​AGA​GG, *MAP2*-F: CAG​GAG​ACA​GAG​ATG​AGA​ATT​CC, *MAP2*-R: CAG​GAG​TGA​TGG​CAG​TAG​AC, *NES*-F: CTG​GAG​CAG​GAG​AAA​CAG​GG, *NES*-R: CCA​GGC​TGA​GGG​ACA​TCT​TG, *PAX6*-F: CCG​TCC​ATC​TTT​GCT​TGG​GA, *PAX6*-R: CTT​TTC​GCT​AGC​CAG​GTT​GC, *SOX2*-F: GCC​GAG​TGG​AAA​CTT​TTG​TCG, *SOX2*-R: GGC​AGC​GTG​TAC​TTA​TCC​TTC​T, *SYN1*-F: CTG​GAC​GTC​CCA​AAC​CAC​AG, *SYN1*-R: GCT​GGC​TCT​GGA​AGG​TTG​AAG, *TUBB3*-F: CCG​GAA​CCA​TGG​ACA​GTG​TCC, *TUBB3*-R: ACC​ACA​TCC​AGG​ACC​GAA​TCC. All data were analyzed using the 2^−ddCT^ method, normalized internally to geometric mean of *EIF4A2*, *ACTB*, and *GAPDH*, and normalized globally to the vehicle treatment groups.

### 2.10 Statistics

Data are expressed as mean ± standard error of mean (SEM). Statistical significance was set at *p* < 0.05. The data were analyzed and graphs were plotted using GraphPad Prism. The difference between the PFOA exposure and control groups was compared using a one-way analysis of variance (ANOVA), followed by Dunnet’s multiple comparisons *post hoc* test. Additionally, differences between sexes were analyzed as a two-way ANOVA with PFOA exposure and sex as factors. When there was a significant effect of dose or an interaction, multiple comparisons were analyzed using Dunnet’s *post hoc* test, compared to control group collapsed across sex and/or control group within sex, respectively. For the initial experiment testing the amount of lipid accumulation after adding PFOA then washing the cells, a one-way ANOVA was performed with each timepoint followed by a Dunnett’s *post hoc* comparing the baseline conditions to PFOA pulse and subsequent washes. The other pulse-chase and efflux experiments were analyzed using a two-way ANOVA with timepoint (i.e., baseline, pulse, chase, *etc.*) and PFOA exposure as factors, followed by a Šidák *post hoc* test to compare the difference between PFOA and CTRL groups within each timepoint. Similarly, the inhibitor experiments were also analyzed using a two-way ANOVA with presence of an inhibitor and exposure as factors, followed by Šidák post hoc test to compare the difference between CTRL and exposure groups with and without the inhibitor. For proliferation IHC experiments, a two-way ANOVA was conducted with timepoint and PFOA exposure as factors, followed by a Dunnett’s post hoc test comparing exposure groups to control group at each timepoint. For the quiescence experiments, a two-way ANOVA was conducted with cell state (i.e., NPC entry) and PFOA exposure as factors, followed by a Dunnett’s post hoc test comparing exposure groups to control group within each cell state. In the lipid peroxidation, mitochondrial ROS and neuronal differentiation experiments, positive controls CHP and Rot and negative control using NPC media, respectively, were analyzed as a two-tailed t-test compared to the vehicle control group. This was performed to not bias the results of the ANOVAs with PFOA exposure as a factor. For calculation of EC_50_, the concentration was log-transformed and nonlinear regression was performed with the Hill slope curve fitting for the EC50 value with comparisons between values analyzed using sum-of-squares F test. Effect size was calculated using Eta squared (η^2^) for the ANOVA and Cohen’s d for *post hoc* testing, and *R* ^2^ was reported for curve-fitting sum-of-squares F tests. For correlation analysis, intensity and gene expression values were normalized as a percentage change from control by dividing the average of the mean values for each control sample across donors then multiplying by 100 then we performed a non-parametric Spearman correlation and calculated a two-tailed t-test between each variable. Additionally, Rstudio was used to perform principal component analysis (PCA). Gene expression data was first standardized using datawizard package and PCA was ran using the stats package and the first to principal components were plotted using ggplot2.

## 3 Results

### 3.1 PFAS cytotoxicity is dependent on chain length, head group and serum supplementation

**FIGURE 1:**
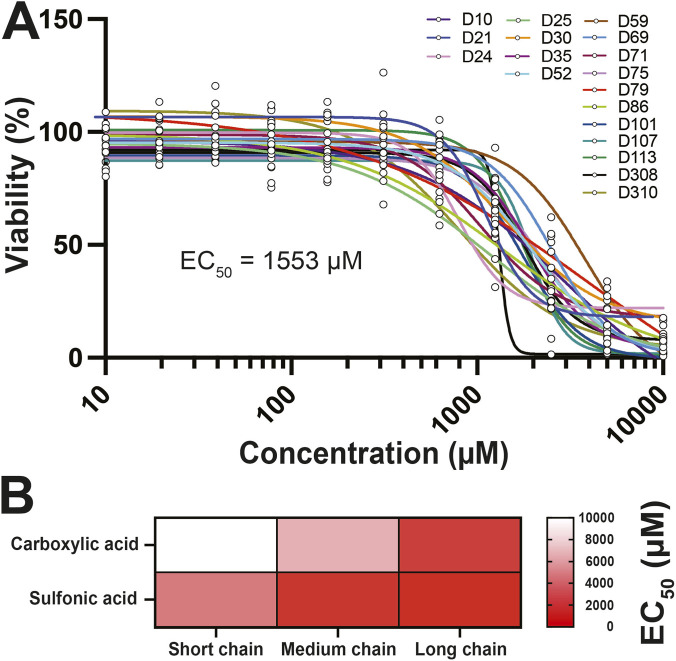
PFOA exposure is cytotoxic to human NPCs in the millimolar range. **(A)** PFOA exposure for 48 h decreased cell viability in human NPCs in a dose-dependent manner (n = 19 donors). **(B)** PFAS chemicals with a longer chain or sulfonate head group had lower EC_50_ values than short chain or carboxylic compounds (n = 3 donors). Nonlinear regression was performed with the Hill slope curve fitting for the EC_50_ value to generate dose-response curves. Each line represents the dose-response curve for each donor line. All values are averages of replicates expressed relative to the viability of the untreated control cells.

We exposed phNPCs to PFOA for 48 h to examine the impact of acute exposure on viability. There was a significant effect of exposure (*F*_10, 187_ = 178.4, *p* < 0.0001, η^2^ = 0.76) on viability but not sex (*F*_1, 187_ = 0.2, *p* = 0.67, η^2^ = 0.00005) or the interaction (*F*_10, 187_ = 0.4, *p* = 0.93, η^2^ = 0.02). Loss of viability was seen at the high micromolar/millimolar range (1,250 μM PFOA: *p* < 0.0001, *d* = 0.63; 2500 μM: *p* < 0.0001, *d* = 1.23; 5,000 μM: *p* < 0.0001, *d* = 1.64; 10000 μM: *p* < 0.0001, *d* = 1.82, Figure 1A). Additionally, we compared the cytotoxicity of other perfluoroalkyl chemicals with similar chemistry in phNPCs, differing only in chain length or functional group. PFAS with sulfonic groups and/or longer chain length had lower EC50 values, suggesting greater cytotoxicity although at millimolar dose ranges (Figure 1B, Supplementary Figure S1A).

Cell death can be initiated by apoptosis, the accumulation of peroxides as seen in ferroptosis, or by external factors in necrosis. We co-exposed phNPCs with PFOA and inhibitors of these pathways (Z-VAD, Fer-1, and Nec-1) to determine if PFOA cytotoxicity could be attenuated. Use of each inhibitor significantly (*F*_3, 216_ = 4.11, *p* < 0.007, *R* ^2^ = 0.92) affected the EC_50_ value, with inhibitor conditions (Nec-1: EC_50_ = 2636 μM; Z-VAD: EC_50_ = 2899 μM; Fer-1: EC_50_ = 2739 μM) having higher EC_50_ values than non-inhibitor condition (EC_50_ = 1990 μM, Supplementary Figure S1B). Removing serum substitute BIT9500 significantly changed the EC_50_ values (*F*_3, 122_ = 4.35, *p* = 0.03, *R* ^2^ = 0.77) resulted in a 10-fold reduction in the EC50 value (+BIT: EC_50_ = 1930 μM, -BIT: EC_50_ = 183 μM) however, it also resulted in a 50% reduction in viability when comparing the vehicle controls between the two media conditions (Supplementary Figure S1C).

### 3.2 PFOA increased lipid accumulation and decreased lipid turnover and fatty acid efflux

**FIGURE 2:**
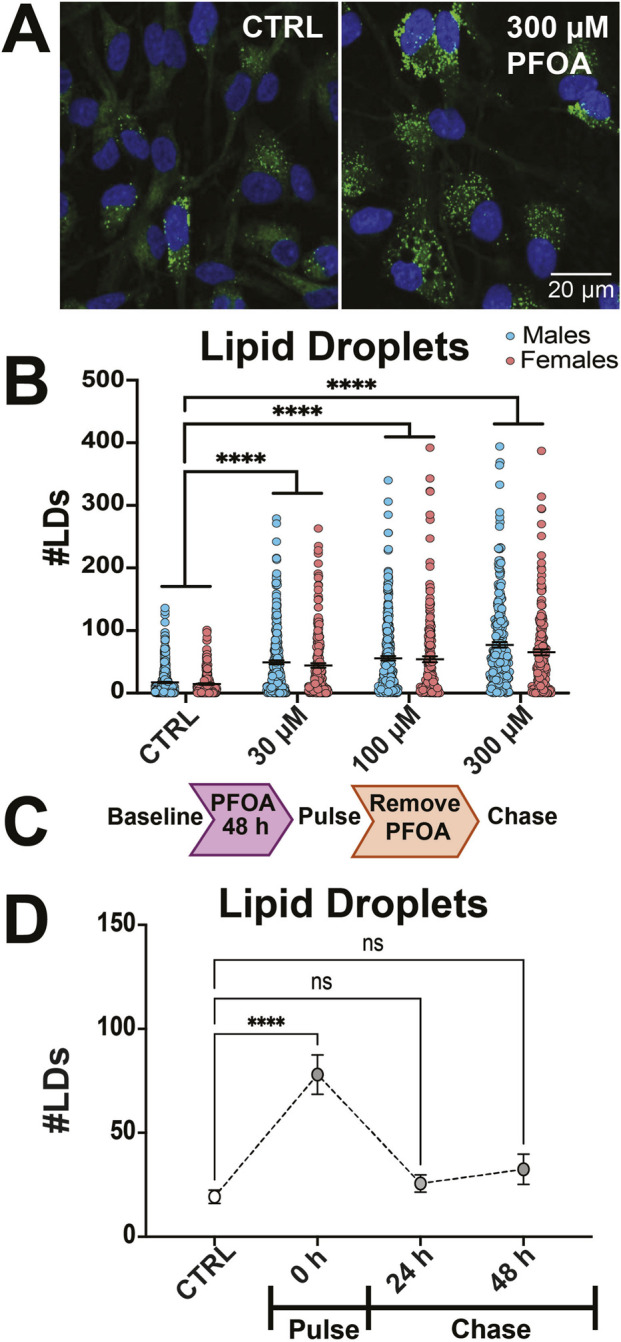
PFOA causes lipid accumulation in human NPCs. **(A)** Representative image of human NPCs exposed to vehicle (CTRL) or 300 µM PFOA for 48 h and stained with BODIPY493 and DAPI. **(B)** Quantification of lipid droplet (LD) number (n = 400–700 cells per condition from 19 donors). **(C)** PFOA pulse chase design and **(D)** LD number quantification (n = 70–100 cells from four donors). Error bars represent mean ± SEM. Two-way ANOVA was conducted with sex and exposure as factors. Dunnett’s *post hoc* test compared to controls collapsed across sex: ****p < 0.0001, n. s = not significant.

PFOA exposure for 48 h resulted in a significant increase in number of LDs (*F*_3, 2068_ = 101.7, *p* < 0.0001, η^2^ = 0.14, Figures 2A,B), BODIPY intensity (*F*_3, 2068_ = 111.1, *p* < 0.0001, η^2^ = 0.15, Supplementary Figure S2A), area covered by LDs (*F*_3, 1891_ = 129.9, *p* < 0.0001, η^2^ = 0.18, Supplementary Figure S2B), and LD size (*F*_3, 1871_ = 37.44, *p* < 0.0001, η^2^ = 0.065, Supplementary Figure S2C), with no effect of sex nor an interaction exposure and sex except for number of LDs where there was a significant effect of sex (*F*_1, 2068_ = 4.79, *p* = 0.03, η^2^ = 0.13). To determine if lipid accumulation occurred after PFOA was removed, phNPCs were treated with PFOA for 48 h, then were washed twice and imaged 24 h and 48 h later (Figure 2C). There was a significant effect of timepoint (number of LDs: *F*_3, 319_ = 7.77, *p* < 0.0001, η^2^ = 0.16; BODIPY intensity: *F*_3, 319_ = 14.86, *p* < 0.0001, η^2^ = 0.12; area covered: *F*_3, 319_ = 19.27, *p* < 0.0001, η^2^ = 0.18, LD size: *F*_3, 319_ = 13.97, *p* < 0.0001, η^2^ = 0.14) with an expected initial increase from 48 h PFOA exposure in the number of LDs (Baseline vs. Pulse: *p* < 0.0001, *d =* 0.81), BODIPY intensity (Baseline vs. Pulse: *p* < 0.0001, *d =* 0.96), area covered (Baseline vs. Pulse: *p* < 0.0001, *d =* 1.01), and LD size (Baseline vs. Pulse: *p* < 0.0001, *d =* 0.97). After washing, the number of LDs (CTRL vs. 24 h Chase: *p* = 0.76, *d =* 0.093; CTRL vs. 48 h Chase: *p* = 0.28, *d =* 0.17), BODIPY intensity (CTRL vs. 24 h Chase: *p* = 0.89, *d =* 0.093; CTRL vs. 48 h Chase: *p* = 0.99, *d =* 0.037), area covered (CTRL vs. 24 h Chase: *p* = 0.99, *d =* 0.0046; CTRL vs. 48 h Chase: *p* = 0.91, *d =* 0.089), and LD size (CTRL vs. 24 h Chase: *p* = 0.99, *d =* 0.047; CTRL vs. 48 h Chase: *p* = 0.81, *d =* 0.12), were not significantly different from baseline at 24 and 48 h post-washing (Figure 2D, Supplementary Figures S2D–F).

**FIGURE 3:**
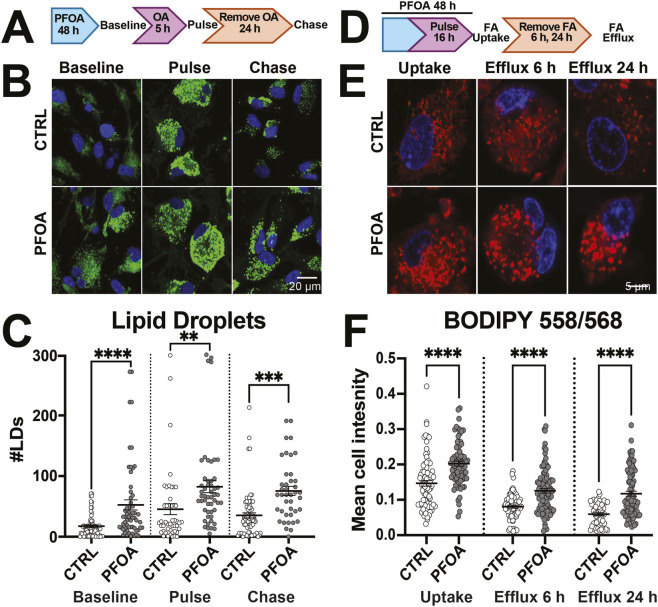
PFOA exposure led to a decrease in lipid turnover and fatty acid efflux and increase in fatty acid uptake. **(A)** NPCs were exposed to vehicle (CTRL) or 300 µM PFOA for 48 h then chased and incubated with 500 µM oleic acid (OA) for 5 h to pulse lipid droplet formation followed by a chase for 24 h to examine lipid turnover. **(B)** Representative image of BODIPY493/DAPI stained human NPCs exposed to CTRL or PFOA then imaged immediately after exposure (Baseline), OA pulse (Pulse), or 24 h after removing OA (Chase). **(C)** PFOA exposure (n = 50–80 cells per condition from four donors) did not alter the amount of lipid droplets after the OA pulse however, once chased PFOA-exposed cells had an increase in the number of lipid droplets compared to vehicle controls. **(D)** NPCs were exposed to vehicle (CTRL) or 300 µM PFOA PFOA for 48 h followed by a 16-h pulse of 1 µM fatty acid analog BODIPY-C12 558/568 to measure uptake then chased for 6 h and 24 h chase to measure fatty acid efflux. **(E)** Representative image of BODIPY 558/568 and Hoechst-33342 stained human NPCs exposed to CTRL or PFOA then imaged immediately after the 16-h BODIPY 558/568 incubation (Uptake), the 6-h chase period (Efflux 6 h) or the 24-h chase (Efflux 24 h). **(F)** PFOA exposure resulted in an increase in cellular BODIPY 558/568 intensity during the Uptake and Efflux (6 h and 24 h) periods compared to controls (CTRL) (n = 100–200 cells per condition from six donors). Error bars represent mean ± SEM. Two-way ANOVA was conducted with exposure and timepoint as factors. Dunnett’s *post hoc* test compared to controls within timepoint: **p < 0.01, ***p < 0.001, ****p < 0.0001.

We performed a pulse chase experiment using oleic acid (OA) to assess lipid processing (Figure 3A). The phNPCs were first exposed to PFOA then washed and treated with a 5 h OA pulse to stimulate LD biogenesis followed by a 24 h chase period where OA was removed from the media during which lipids were metabolized (Figure 3A). There was a significant effect of PFOA exposure and OA-pulse/chase timepoint on number of LDs (Exposure: *F*_1, 345_ = 45.2, *p* < 0.0001, η^2^ = 0.11; Timepoint: *F*_2, 345_ = 10.06, *p* < 0.0001, η^2^ = 0.050), BODIPY intensity (Exposure: *F*_1, 300_ = 30.77, *p* < 0.0001, η^2^ = 0.076; Timepoint: *F*_2, 300_ = 37.43, *p* < 0.0001, η^2^ = 0.18), area covered (Exposure: *F*_1, 316_ = 23.85, *p* < 0.0001, η^2^ = 0.049; Timepoint: *F*_2, 316_ = 73.49, *p* < 0.0001, η^2^ = 0.30), and LD size (Exposure: *F*_1, 329_ = 22.88, *p* < 0.0001, η^2^ = 0.047; Timepoint: *F*_2, 329_ = 65.32, *p* < 0.0001, η^2^ = 0.27) (Figures 3B,C).

After the OA-pulse, PFOA-exposed cells had a significant increase in number of LDs (*p* = 0.0011, *d =* 0.43, Figure 3C), BODIPY intensity (*p* = 0.0013, *d =* 0.38, Supplementary Figure S3A) and LD size (*p* = 0.007, *d =* 0.32, Supplementary Figure S3B), suggesting increased lipid synthesis compared to controls. Additionally, after the chase period, PFOA-treated cells still had a higher number of LDs (*p* < 0.0001, *d =* 0.42), BODIPY intensity (*p* = 0.03, *d =* 0.28), area covered *p* = 0.0004, *d =* 0.41), and LD size (*p* = 0.0038, *d =* 0.35), suggesting decreased lipid turnover.

We also examined how PFOA exposure impacts the ability to transport fatty acids using a labelled fatty acid analog BODIPY558/568 (Figure 3D). There was a significant effect of PFOA exposure (*F*_1, 464_ = 91.68, *p* < 0.0001, η^2^ = 0.12) and timepoint (*F*_2, 464_ = 93.68, *p* < 0.0001, η^2^ = 0.25) on BODIPY 558/568 intensity. PFOA exposure resulted in an increase in the initial intensity of BODIPY after the pulse (*p* < 0.0001, *d =* 0.55) and a continued higher signal intensity during the chase period (6 h: *p* < 0.0001, *d =* 0.45, 24 h: <0.0001, *d =* 0.53), suggesting an increase in influx and decrease in efflux of fatty acids (Figures 3E,F).

### 3.3 PFOA increased lipid peroxidation, reactive oxygen species and altered mitochondrial morphology

**FIGURE 4:**
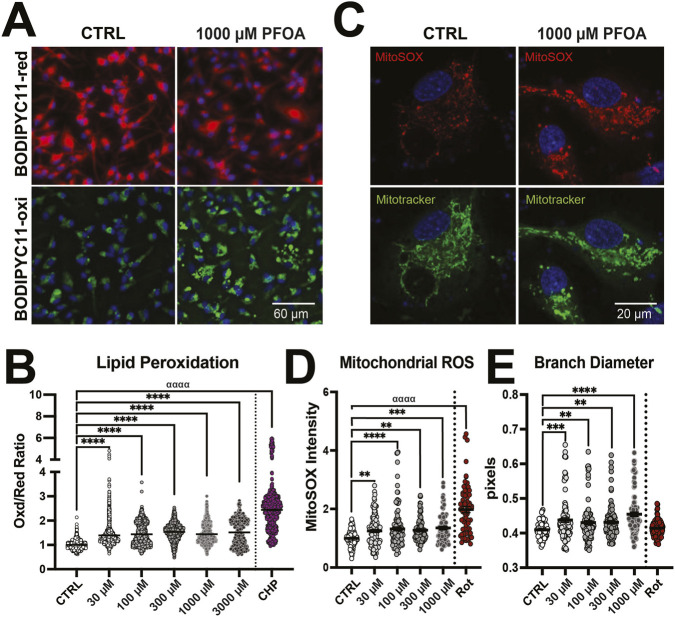
PFOA exposure increased lipid peroxidation and mitochondrial reactive oxygen species (ROS). **(A)** Representative image of human NPCs exposed to vehicle (CTRL) or 1,000 µM PFOA for 4 h then treated with 5 µM lipid peroxide sensor BODIPY-C11 581/591 with the reduced form of BODIPYC11(BODIPYC11-red) in red and the oxidized form (BODIPYC11-oxi) in green and Hoechst-33342-stained nuclei in blue. **(B)** Exposure to 30–3,000 µM PFOA and positive control 50 µM cumene hydroxide (CHP) for 4 h resulted in an increase in the ratio of oxidized to reduced form of BODIPYC11 compared to vehicle controls. (n = 600–1,400 cells per condition from eight donors). **(C)** Representative image of human NPCs exposed to vehicle (CTRL) or 1,000 µM PFOA for 48 h then stained with mitochondrial ROS sensor MitoSOX, mitochondrial marker Mitotracker, and Hoechst-33342. **(D)** Exposure to 30–1,000 µM PFOA (n = 70–130 cells per condition from six donors) or 10 µM rotenone (Rot) resulted in an increase in cellular MitoSOX intensity, compared to vehicle controls (CTRL). **(E)** Exposure to 30–3,000 µM PFOA also resulted in a decrease in mitochondrial diameter. Error bars represent mean ± SEM. One-way ANOVA was conducted and a Dunnett’s post hoc test compared to controls collapsed across sex: **p < 0.01, ***p < 0.001, ****p < 0.0001. A two-tailed t-test comparing CTRL to positive controls (CHP) or (Rot): ^αααα^p<0.0001.

A consequence of excess lipid accumulation is cellular damage. We assessed whether PFOA exposure resulted in lipid peroxidation using BODIPY 581/591 C11 (BODIPY-C11) which makes a fluorescent shift from an emission peak at 590 nm (red) to 510 nm (green) following oxidation. We observed a significant effect of PFOA exposure (*F*_6, 8608_ = 1,006, *p* < 0.0001, η^2^ = 0.41), which increased oxidized lipids across all PFOA doses (30 μM: *p* < 0.0001, *d =* 0.46; 100 μM: *p* < 0.0001, *d =* 0.51; 300 μM: *p* < 0.0001, *d =* 0.62; 1,000 μM: *p* < 0.0001, *d =* 0.45; 1,000 μM: *p* < 0.0001, *d =* 0.45) (Figures 4A,B). Cells can remove excess lipids *via* metabolism in mitochondria using beta-oxidation. Using the MitoSOX probe to measure mitochondrial reactive oxygen species (ROS), there was a significant effect of PFOA exposure on MitoSOX intensity (*F*_5, 569_ = 26.19, *p* < 0.0001, η^2^ = 0.19) with PFOA exposure increasing MitoSOX intensity (30 μM: *p* = 0.004, *d =* 0.28; 100 μM: *p* < 0.0001, *d =* 0.37; 300 μM: *p* = 0.002, *d =* 0.30; 1,000 μM: *p* < 0.0001, *d =* 0.37) (Figures 4C,D). Excessive ROS can also alter mitochondrial function. We therefore examined mitochondrial morphology in treated and untreated cells. There was a significant effect of PFOA exposure on the compactness (*F*_5, 573_ = 12.43, *p* < 0.0001, η^2^ = 0.098, Supplementary Figure S3D), branch length (*F*_5, 565_ = 9.50, *p* < 0.0001, η^2^ = 0.078, Fig. S3E), branch diameter (*F*_5, 554_ = 9.42, *p* < 0.0001, η^2^ = 0.078, Figure 4E), and mitochondrial footprint (*F*_5, 547_ = 2.26, *p* < 0.04, η^2^ = 0.068, Supplementary Figure S3F). Unlike the control cells which displayed a fused network of mitochondria, mitochondria from PFOA-treated cells were more compact (30 μM: *p* = 0.02, *d* = 0.24; 300 μM: *p* = 0.03, *d* = 0.23; 1,000 μM: *p* < 0.0001, *d* = 0.42, Supplementary Figure S3D), shorter (300 μM: *p* = 0.03, *d* = 0.25; 1,000 μM: *p* < 0.0001, *d* = 0.50, S3E), and thicker (30 μM: *p* = 0.0002, *d* = 0.32; 100 μM: *p* = 0.006; 300 μM: *p* = 0.003, *d* = 0.25; 1,000 μM: *p* < 0.0001, *d* = 0.50, Figure 4E), suggesting more fragmented mitochondria from cellular stress.

### 3.4 PFOA-induced lipid accumulation is dependent on autophagy and lipolysis

**FIGURE 5:**
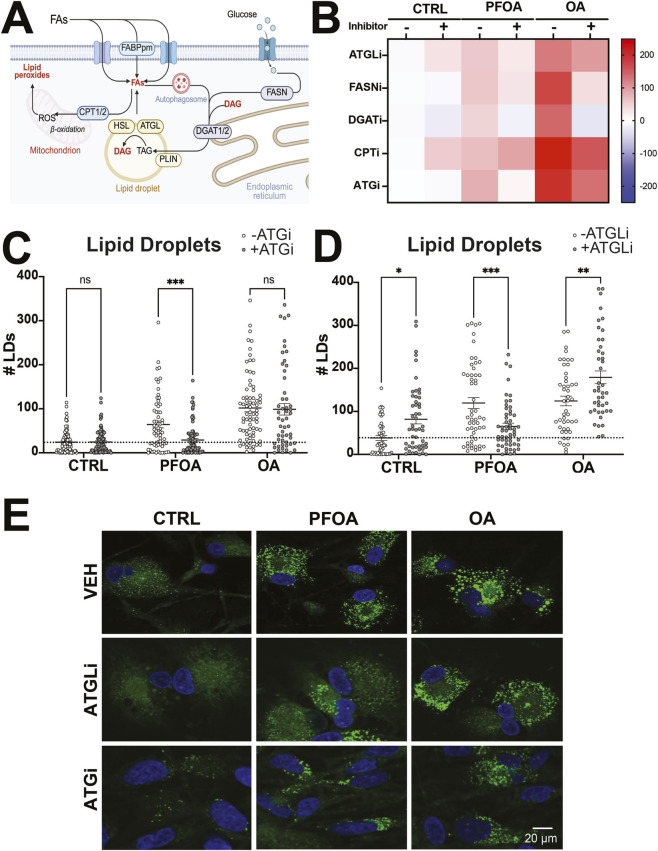
PFOA-associated lipid accumulation is dependent upon lipolysis and autophagy. **(A)** Diagram of lipid metabolism pathways. **(B–E)** Human NPCs were exposed to vehicle (CTRL) or 300 µM PFOA and lipid metabolism inhibitors for lipoplysis (ATGLi), fatty acid synthase (FASNi), diglyceride acyltransferase (DGATi), fatty acid oxidation (CPTi) and autophagy (ATGi) for 48 h and stained with BODIPY493 for lipid droplets (n = 100–200 cells per condition from four donors). **(B)** Heatmap of BODIPY intensity normalized to control group with no inhibitors. **(C)** Exposure to lipolysis inhibitor Atglistatin (ATGLi) lead to an increase in lipid droplet numbers in control- and OA-treated NPCs but a decrease in PFOA-exposed NPCs. **(D)** Exposure to autophagy inhibitor 3-methyladenine (ATGi) resulted in a reduction in lipid droplet number only in PFOA-treated NPCs with no effects in control or OA-treated cells. Representative image of human NPCs exposed to vehicle (CTRL) or 300 µM PFOA for 48 h and stained with BODIPY493 and DAPI. **(E)** Representative image of human NPCs exposed to vehicle (CTRL) or 300 µM PFOA for 48 h and co-treated with Atglistatin (ATGLi), 3-methyladenine (ATGi), or vehicle (VEH) and stained with BODIPY493 and DAPI. Error bars represent mean ± SEM. Two-way ANOVA was conducted with exposure and inhibitor as factors. Dunnett’s post hoc test compared the difference between CTRL and exposure groups with and without the inhibitor: *p < 0.05, **p < 0.01, ***p < 0.001, n. s = not significant. Abbreviations: ATG: autophagy; ATGL: adipose triglyceride lipase; CPT1/2, carnitine palmitoyltransferase-1/2; DAG, diacylglycerol; DGAT1/2, Diglyceride acyltransferase-1/2; FABPpm, fatty acid binding protein; FAs, fatty acids; FASN, fatty acid synthase; HSL, hormone-sensitive lipase; PLIN, Perilipin; ROS, reactive oxygen species TAG, triglyceride.

The formation of lipid droplets is a tightly regulated cellular process that involves the import or synthesis of fatty acids, incorporation into a lipid droplet, lipolysis and further breakdown *via* beta oxidation (Figure 5A). We sought to investigate which processes may enhance or suppress PFOA-associated LD formation. Using a variety of inhibitors, we co-exposed cells to vehicle, PFOA, or OA for 48 h, then stained with BODIPY (Figure 5B, Supplementary Figure S5).

Inhibition of fatty acid synthesis (Supplementary Figures S4A–D) resulted in a significant effect of exposure (*F*_2, 431_ = 33.76, *p* < 0.0001, η^2^ = 0.12) and FASNi (*F*_1, 431_ = 71.35, *p* < 0.0001, η^2^ = 0.12) or the interaction (*F*_2, 431_ = 2.93, *p* = 0.06, η^2^ = 0.10) on the number of LDs with FASNi addition resulting in a decrease in the number of LDs in CTRL- (*p* = 0.003, *d =* 0.32), PFOA- (*p* < 0.0001, *d =* 0.52) or OA-treated (*p* < 0.0001, *d =* 0.56), suggesting that FASNi can modulate the number of LDs in phNPCs but PFOA-associated lipid accumulation is not dependent on fatty acid synthesis. There was also a significant effect of exposure (BODIPY: *F*_2, 431_ = 103.0, *p* < 0.0001, η^2^ = 0.29), inhibitor (BODIPY: *F*_2, 431_ = 34.36, *p* < 0.0001, η^2^ = 0.049), and the interaction (BODIPY: *F*_2, 431_ = 14.23, *p* < 0.0001, η^2^ = 0.041) on BODIPY intensity and area covered (Area: *F*_2, 431_ = 13.41, *p* < 0.0001, η^2^ = 0.010) but only OA-treated cells (BODIPY: *p* < 0.0001, *d =* 0.72; Area: *p* = 0.0002, *d =* 0.39) had significant reductions.

The inhibition of fatty acid breakdown by blocking Cpt1a (Supplementary Figures S4M–P) resulted in a significant effect of the interaction of PFOA/OA exposure and inhibitor on number of LDs (*F*_2, 406_ = 16.81, *p* < 0.0001, η^2^ = 0.056), BODIPY intensity (*F*_2, 393_ = 37.79, *p* < 0.0001, η^2^ = 0.097), area covered (*F*_2, 382_ = 8.24, *p* = 0.0003, η^2^ = 0.030), and LD size (*F*_2, 382_ = 3.47, *p* = 0.03, η^2^ = 0.016).

Specifically, blocking Cpt1a resulted in an increase in LDs in OA-treated (*p* < 0.0001, *d =* 0.58), BODIPY intensity in CTRL- (*p* < 0.0001, *d =* 0.69) and OA-treated (*p* < 0.0001, *d =* 0.55), and area covered and LD size in the CTRL-treated (Area: *p* = 0.0003, *p* = 0.03, *d =* 0.25) phNPCs, suggesting that blocking beta oxidation resulted in an increase in lipid accumulation in CTRL- and OA-treated but not PFOA-treated cells.

The rate limiting step of formation of LDs is controlled by DGAT proteins. There was a significant effect of DGAT inhibitors (Supplementary Figures S4Q–T) on number of LDs (*F*_6,1530_ = 233.0, *p* < 0.0001, η^2^ = 0.29), BODIPY intensity (*F*_6,1530_ = 407.3, *p* < 0.0001, η^2^ = 0.33), area covered (*F*_6,1435_ = 294.0 *p* < 0.0001, η^2^ = 0.32), and LD size (*F*_6,1271_ = 82.42, *p* < 0.0001, η^2^ = 0.15). Regardless of exposure, lipid accumulation was greatly diminished in cells treated with either DGAT inhibitor; with DGAT1i resulting in complete removal of LDs.

Treatment with autophagy inhibitor 3-MA resulted in an interaction between 3-MA and treatment on number of LDs (*F*_2,458_ = 5.07, *p* = 0.007, η^2^ = 0.016, Figures 5C,E), BODIPY intensity (*F*_2,458_ = 15.96, *p* < 0.0001, η^2^ = 0.031, Supplementary Figure S5J), and area covered (*F*_2,458_ = 7.75, *p* = 0.0005, η^2^ = 0.023, Supplementary Figure S4K) but not LD size (*F*_2,428_ = 2.978, *p* = 0.04, η^2^ = 0.012, Supplementary Figure S5L). Co-treatment with 3-MA resulted in a reduction in LDs in PFOA-treated cells but not CTRL- or OA-treated, suggesting PFOA-associated lipid accumulation may be dependent on autophagy.

Inhibition of lipolysis using ATGL resulted in a significant interaction of ATGL and PFOA/OA-treatment on number of LDs (*F*_2,291_ = 16.36, *p* < 0.0001, η^2^ = 0.082, Figures 5D,E), BODIPY intensity (*F*_2,299_ = 14.72 *p* < 0.0001, η^2^ = 0.063, Supplementary Figure S4F), area covered (*F*_2,299_ = 6.10, *p* = 0.003, η^2^ = 0.026, Supplementary Figure S5G), and LD size (*F*_2,299_ = 3.84, *p* = 0.02, η^2^ = 0.020, Supplementary Figure S5H). Interestingly, inhibition of lipolysis using ATGL resulted in an increase in LDs (CTRL: *p* = 0.01, *d =* 0.35; OA: *p* = 0.002, *d =* 0.41) and BODIPY intensity (CTRL: *p* = 0.01, *d =* 0.31; OA: *p* = 0.002, *d =* 0.29) in CTRL- and OA-treated but a decrease in LDs (*p* = 0.0006, *d =* 0.44) and BODIPY intensity (*p* = 0.0002, *d =* 0.48) in PFOA-treated cells, suggesting that LDs from PFOA-associated cells may be broken down by an alternative process.

### 3.5 PFOA exposure had limited effect on NPC proliferation

**FIGURE 6:**
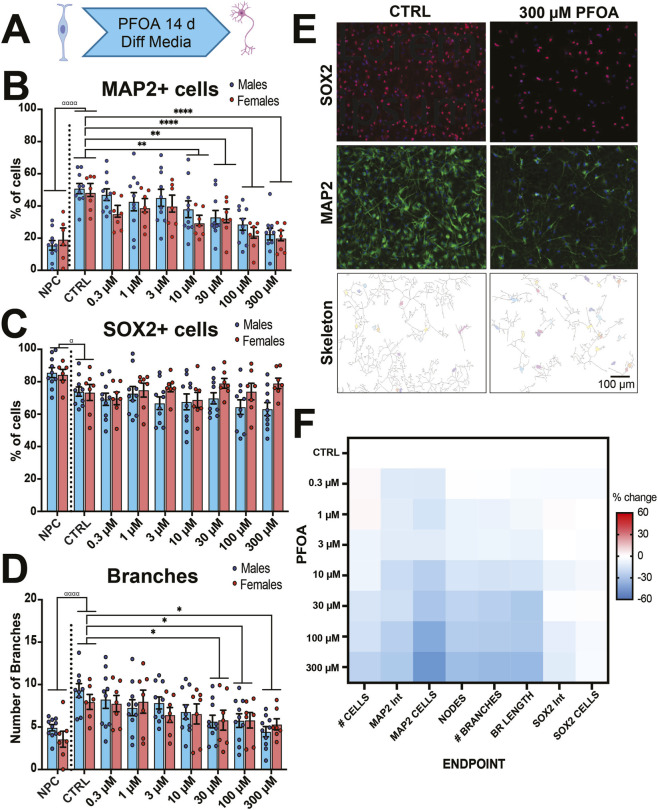
PFOA exposure during neuronal differentiation resulted in a reduction in neurons and decrease in number of neuronal branches. **(A)** Human NPCs (n = 16 donors) were exposed to PFOA or vehicle (CTRL) for 14 days during neuronal differentiation. A set of wells in maintained in NPC media (NPC) served as a control to compare neuronal differentiation. All wells were fixed and stained at the end of the timepoint. NPCs exposed to 10–300 µM PFOA resulted in a reduction in MAP2+ cells **(B)** but not SOX2+ cells **(C)** compared to control cells (CTRL). **(D)** PFOA exposure (30–300 µM) also altered neuronal morphology, creating neurons with few branches. **(E)** Representative image of SOX2 and MAP2 staining and skeletonized neurons of differentiated neurons treated with vehicle (CTRL) or 300 µM PFOA. **(F)** Summary heatmap of phenotype changes, normalized to percentage change relative to control. Error bars represent mean ± SEM. Two-way ANOVA was conducted with sex and exposure as factors. Dunnett’s post *hoc* test compared to controls collapsed across sex: *p < 0.05, **p < 0.01, ***p < 0.001, ***p < 0.0001. A two-tailed t-test comparing CTRL to negative control using NPC Media controls (NPC): ^α^p<0.05, ^αααα^p<0.0001.

Previous work from our lab showed that PFOA exposure for 4 days *in vivo* altered the proportion of neuronal progenitor cells (NPCs) and neurons in the mouse brain. To determine if similar effects occur in human cells, we investigated the impact of PFOA on phNPCs *in vitro* (Figure 6A). We first exposed phNPCs to non-cytotoxic doses of PFOA for 4 days, followed by EdU incorporation to quantify the proportion of proliferating cells. We did not see a significant effect of exposure, sex nor an interaction on the number of cells in G2/M (PFOA: *F*_7,75_ = 0.53, *p* = 0.81, η^2^ = 0.044; Sex: *F*_1,75_ = 0.061, *p* = 0.81, η^2^ = 0.0007; PFOA × Sex: *F*_1,75_ = 0.87, *p* = 0.53, η^2^ = 0.072), G1/G0 (PFOA: *F*_7,75_ = 0.56, *p* = 0.79, η^2^ = 0.046; Sex: *F*_1,75_ = 0.41, *p* = 0.52, η^2^ = 0.0050; PFOA × Sex: *F*_1,75_ = 0.84, *p* = 0.55, η^2^ = 0.069), or S phase (PFOA: *F*_7,75_ = 0.65, *p* = 0.65, η^2^ = 0.061; Sex: *F*_1,75_ = 0.08, *p* = 0.77, η^2^ = 0.0010; PFOA × Sex: *F*_1,75_ = 0.47, *p* = 0.85, η^2^ = 0.040) (Supplementary Figures S6E,F). We followed up imaging S phase marker EdU, mitosis marker pHH3 and proliferative marker Ki67 to look for changes in cell cycle over time (Supplementary Figures S4A–D). While there was no effect of exposure (*F*_4,155_ = 1.737, *p* = 0.14, η^2^ = 0.041), timepoint (*F*_3,155_ = 1.388, *p* = 0.25, η^2^ = 0.024), nor an interaction (*F*_3,155_ = 0.36, *p* = 0.97, η^2^ = 0.026) on pHH3+ cells at any time points (Figure 6F), we did observe a significant effect of exposure on Ki67+ cells (*F*_4,160_ = 3.55, *p* = 0.008, η^2^ = 0.080, Figure 6D) and EdU + cells (*F*_4,157_ = 3.99, *p* = 0.004, η^2^ = 0.088, Figure 6E) with a reduction of these markers at later time points only at the highest dose.

An alternative explanation for the observed shift in the proportion of NPCs to neurons is that PFOA induces a transition of NPCs from an actively proliferating state into quiescence. To test this hypothesis, we exposed phNPCs to PFOA under three conditions: (1) in proliferative media, (2) in quiescent medium, and (3) during transitions between these two states (i.e., exiting or entering proliferation). These experiments allowed us to assess the effects of PFOA on actively proliferating cells, quiescent cells, and cells that are undergoing state transitions (Supplementary Figure S6g). Neither PFOA exposure nor the interaction with cell state and PFOA exposure had any effect on the number of pHH3+ cells (PFOA: *F*_4,100_ = 2.287, *p* = 0.07, η^2^ = 0.062; PFOA × State: *F*_12,100_ = 0.81, *p* = 0.64, η^2^ = 0.046, Supplementary Figure S6H) or on the number of Ki67+ cells (PFOA: *F*_4,100_ = 0.76, *p* = 0.55, η^2^ = 0.093; PFOA × State: *F*_12,100_ = 0.20, *p* = 0.99, η^2^ = 0.064, Supplementary Figure S6I), suggesting shifting into cellular quiescence is not involved in change in number proportion of NPCs to neurons.

### 3.6 PFOA exposure reduced the number of neurons during differentiation

We also examined the impact of PFOA exposure on neuronal differentiation. NPCs were differentiated for 14 days while being exposed to PFOA or vehicle control. On day 14, we examined the neuronal marker MAP2, the neuronal stem cell marker SOX2, and neuronal morphology (Figures 6B–D). There was a significant effect of dose (*F*_7,120_ = 11.97, *p* < 0.0001, η^2^ = 0.39) and sex (*F*_1,120_ = 4.54, *p* = 0.03, η^2^ = 0.018) but no interaction (*F*_7,120_ = 0.53, *p* = 0.83, η^2^ = 0.017) on the percentage of MAP2+ cells (Figure 6B) with exposure to PFOA resulting in a decrease in MAP2+ cells (10 μM PFOA: *p* = 0.003; *d* = 0.63, 30 μM: *p* = 0.001, *d* = 0.67; 100 μM: *p* < 0.0001 *d*ays = 0.97; 300 μM: *p* < 0.0001, *d* = 1.12). However, there was no effect of PFOA exposure (*F*_7,120_ = 0.65, *p* = 0.71, η^2^ = 0.12) or the interaction of sex and exposure (*F*_7,120_ = 1.01, *p* = 0.43, η^2^ = 0.13) on the percentage of SOX2+ cells (Figure 6C).

**FIGURE 7:**
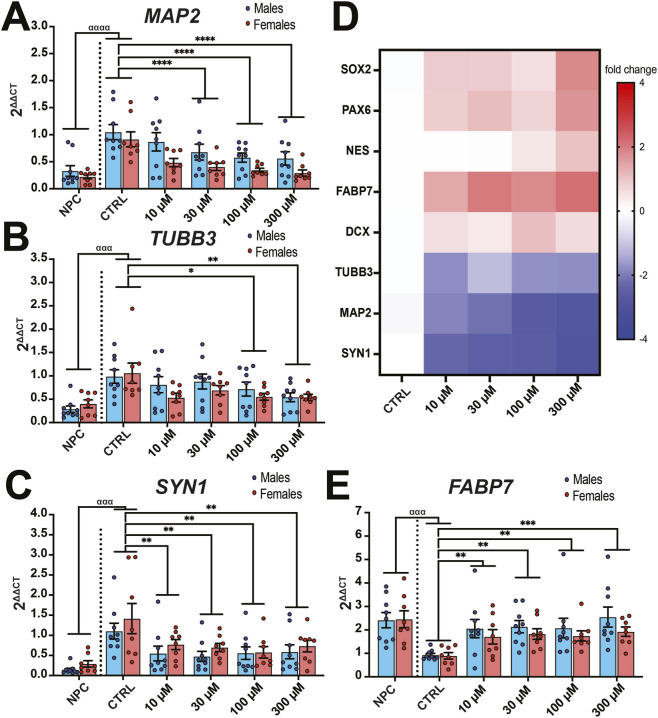
PFOA exposure reduced the expression of neuronal markers and increased the expression of neuronal progenitor markers. Human NPCs (n = 17 donors) were maintained in NPC media (NPC) or changed to neuronal differentiation media and treated with 10–300 µM PFOA exposure or vehicle (CTRL) for 14 days. Exposure to PFOA resulted in a reduction in gene expression in neuronal markers **(A)** *MAP2*, **(B)** *TUBB3*, **(C)** *SYN1* but increased gene expression of NPC/radial glia marker **(E)** *FABP7*. **(D)** Heatmap of gene expression measured in fold change normalized to vehicle controls. Error bars represent mean ± SEM. Two-way ANOVA was conducted with sex and exposure as factors. Dunnett’s post hoc test compared to controls collapsed across sex: *p < 0.05, **p < 0.01, ***p < 0.001, ****p < 0.0001. A two-tailed t-test comparing CTRL to negative control using NPC Media controls (NPC): ^ααα^p<0.001, ^αααα^p<0.0001.

Changes in percentages of MAP2+ or SOX2+ cells were not due to the number of DAPI + cells (Exposure: *F*_7,120_ = 1.23, *p* = 0.29, η^2^ = 0.26). Instead, PFOA exposure led to reductions in SOX2+MAP2+, SOX2-MAP2+ and increases in cells stained for neither SOX2-MAP2- (Supplementary Figure S7A,B). PFOA exposure but not sex nor the interaction of sex and exposure had a significant effect on number of branch nodes (*F*_7,120_ = 5.30, *p* < 0.0001, η^2^ = 0.23, Supplementary Figure S7c), branch length (*F*_7,120_ = 5.30, *p* < 0.0001, η^2^ = 0.20, Supplementary Figure S7D), and number of branches (*F*_7,120_ = 4.30, *p* < 0.0001, η^2^ = 0.23, Figure 7D) with exposure to PFOA resulting in a reduction in all measurements at the higher doses, suggesting neurons with less branching structures. Overall, PFOA exposure reduced the number and branching morphology of neurons (Figures 6E,F).

**FIGURE 8:**
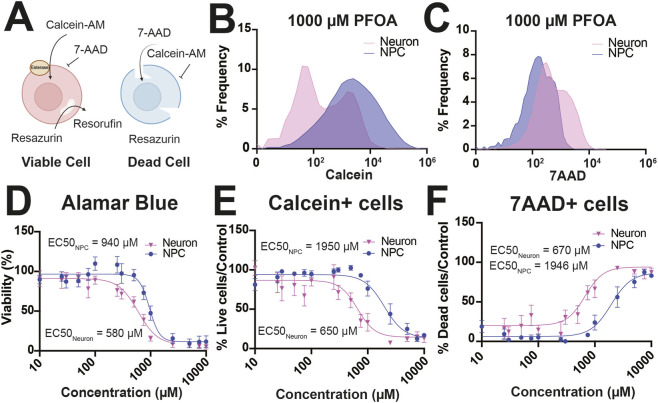
Neurons are more sensitive to PFOA exposure than NPCs. **(A)** Human differentiated neurons or NPCs (n = 6 donors) were exposed to PFOA (10,000–10 µM) for 14 days and assessed for viability using resazurin reducing Alamar blue assay, number of live and dead cells using Calcein-AM and 7-Aminoactinomycin D (7-AAD) staining, respectively. **(B and C)** Representative histograms of **(B)** Calcein-AM or **(C)** 7-AAD signal after 1,000 µM PFOA in neurons and NPCs. Neurons had higher EC_50_ values compared to NPCs using the **(D)** Alamar blue viability assay, **(E)** number of Calcein-AM+ cells, **(F)** number of 7-AAD+ cells. Error bars represent mean ± SEM. Each line represents the dose-response curve for neurons or NPCs. All values are averages of replicates expressed relative to the viability of the untreated control cells for each donor line.

While there was no effect of PFOA exposure on gene expression of progenitor markers *SOX2* (*F*_4,75_ = 1.321, *p* = 0.27, η^2^ = 0.37, Supplementary Figure S7E), *NES* (*F*_4,75_ = 1.93, *p* = 0.11, η^2^ = 0.14, Supplementary Figure S7H), or *PAX6* (*F*_4,75_ = 1.00, *p* = 0.41, η^2^ = 0.21, Supplementary Figure S7F), there was a significant effect of exposure on *FABP7* (*F*_4,75_ = 5.83, *p* = 0.0004, η^2^ = 0.30, Figure 7E), with PFOA exposure increasing expression (10 μM: *p* = 0.03, *d =* 0.57; 30 μM: *p* = 0.0007, *d =* 0.84; 100 μM: *p* < 0.0001, *d =* 0.99; 300 μM: *p* < 0.0001, *d =* 1.05). Neuronal markers *TUBB3* (*F*_4,75_ = 3.66, *p* = 0.009, η^2^ = 0.25, Figure 7B), and *SYN1* (*F*_4,75_ = 4.86, *p* = 0.002, η^2^ = 0.23, Figure 7C) were significantly reduced in expression by PFOA exposure across multiple doses (*TUBB3*: 100 μM: *p* = 0.02, *d =* 0.64; 300 μM: *p* = 0.002, *d =* 0.79; *SYN1*: 10 μM: *p* = 0.003, *d =* 0.74; 30 μM: *p* = 0.0008, *d =* 0.83; 100 μM: *p* = 0.0005, *d =* 0.85; 300 μM: *p* = 0.004, *d =* 0.73), while immature neuron/progenitor marker *DCX* (*F*_4,75_ = 0.76, *p* = 0.55, η^2^ = 0.13, Supplementary Figure S7G) was unchanged after PFOA exposure. Finally, there was a significant effect of sex (*F*_1,75_ = 12.52, *p* = 0.0007, η^2^ = 0.018) and dose (*F*_4,75_ = 7.55, *p* < 0.0001, η^2^ = 0.33) but not an interaction (*F*_4,75_ = 0.30, *p* = 0.88, η^2^ = 0.059) on *MAP2* gene expression, with a reduction in *MAP2* expression across all doses (10 μM: *p* = 0.03, *d =* 0.58; 30 μM: *p* = 0.0007, *d =* 0.84; 100 μM: *p* < 0.0001, *d =* 0.99; 300 μM: *p* < 0.0001, *d =* 1.05, Figure 7A), PCA was performed on the gene expression data to compare vehicle-treated neurons (VEH), PFOA-treated neurons (PFOA), and undifferentiated phNPCs (NPC) which were not in neuronal media (Figure 8A). The VEH and NPC groups segregated along the first two components, which accounted for 45.33% and 24.59% of the variance, respectively. In contrast, the PFOA-group occupied an intermediate position between the VEH and NPC clusters, consistent with an intermediate gene expression profile between neurons and undifferentiated NPCs. Additionally, by leveraging phNPC lines from multiple donors across assays, we examined correlations between key endpoints. Notably, BODIPY intensity after 48 h of PFOA exposure showed a negative correlation with both MAP2 protein levels (r = −0.25, p = 0.06, Figure 8B) and MAP2 gene expression (*r* = −0.37, p = 0.007, Figure 8C) measured after 14 days of neuronal differentiation. These findings indicate that donor lines exhibiting greater lipid droplet accumulation early in PFOA exposure were associated with reduced neuronal differentiation at later time points.

### 3.7 Neurons are more sensitive to PFOA cytotoxicity than NPCs

**FIGURE 9:**
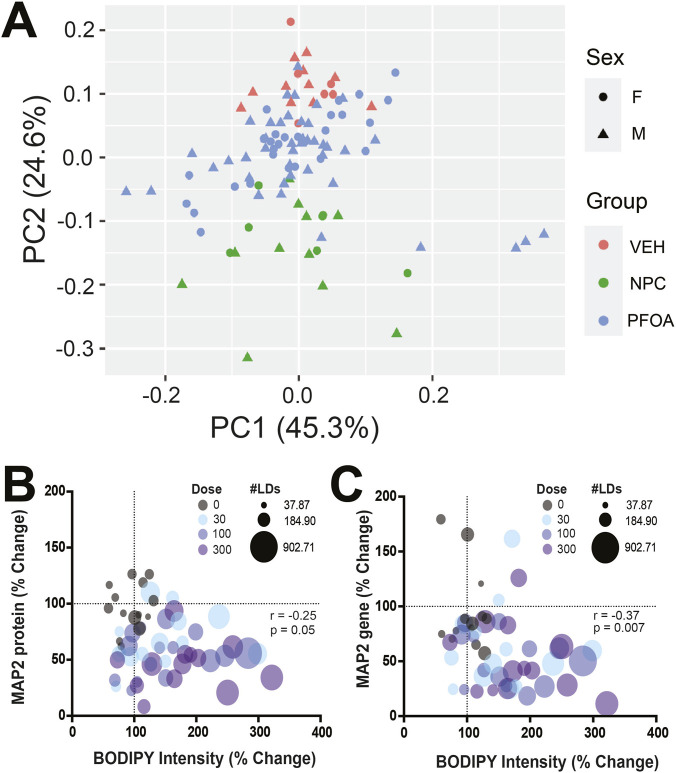
MAP2 gene and protein expression are associated with BODIPY intensity in PFOA-treated donor cells. **(A)** Human NPCs (n = 14 donors) were maintained in NPC media (NPC) or changed to neuronal differentiation media and treated with 10–300 µM PFOA exposure or vehicle (CTRL) for 14 days. PCA plot of gene expression shows clustering of PFOA-treated cells in between NPC and CTRL neurons. **(B and C)** Gene expression of *MAP2* **(B)** and protein expression of MAP2 **(C)** are negatively correlated with BODIPY intensity (n = 14 donors). Values for MAP2 gene and protein expression and BODIPY intensity were normalized to vehicle-treated controls (CTRL) and represented as a percentage change from controls. Corresponding values for each donor line were plotted on the graphs and Spearman correlation was calculated.

The observed reduction in neurons following PFOA exposure could result from either direct impairment of neuronal differentiation and/or increased susceptibility of differentiated neurons to the cytotoxic effects of PFOA. To distinguish between these possibilities, we compared the cytotoxicity of PFOA in undifferentiated phNPCs *versus* neurons differentiated for 5 weeks, following 14 days of exposure (Figure 9A). Neurons exhibited significantly greater sensitivity to PFOA than NPCs across multiple viability and cytotoxicity readouts. In the Alamar Blue assay, neurons displayed a lower EC_50_ (*F*_1, 194_ = 12.99, *p* < 0.0004, *R* ^2^ = 0.79, EC50_Neuron_ = 580 μM, EC50_NPC_ = 940 μM, Figure 9D). Similarly, the proportion of Calcein+ (viable) cells showed a lower EC_50_ in neurons than NPCs (*F*_1, 194_ = 15.81, *p* < 0.0001, *R* ^2^ = 0.72, EC50_Neuron_ = 650 μM, EC50_NPC_ = 1950 μM Figures 9B,E), while the proportion of 7AAD+ (dead) cells confirmed greater sensitivity in neurons (*F*_1, 194_ = 16.29, *p* < 0.0001, *R* ^2^ = 0.74, EC50_Neuron_ = 670 μM, EC50_NPC_ = 1,946 μM Figures 9C,F). Although these EC_50_ values for neurons are in the high micromolar range, these results indicate that differentiated neurons are more vulnerable to prolonged PFOA exposure than undifferentiated NPCs.

## 4 Discussion

We found that PFOA exhibited low acute cytotoxicity in phNPCs, consistent with studies in neuronal-like cells including neuroblastoma (Lagostena et al., 2024), SH-SY5Y (Souders et al., 2021; Running et al., 2024), mouse cortical neurons (Ko et al., 2024), and rat neurons and stem cells (Pierozan and Karlsson, 2021). PFAS with longer-chain length and sulfonic head groups showed greater toxicity, aligning with higher brain accumulation of long-chain PFAS in a variety of species (Van de Vijver et al., 2007; Greaves et al., 2013; Wang et al., 2018; Dassuncao et al., 2019). PFAS containing a sulfonic group or long chain are more detrimental to neurodevelopment than ones with a carboxylic group or short chain (Liao et al., 2009; Ulhaq et al., 2013; Gaballah et al., 2020; Menger et al., 2020). Although the mechanism is not fully understood, it has been suggested that increased lipophilicity of the longer-chains and the polar-charge of the sulfonic group allow these PFAS to more readily incorporate into lipid membranes (Jing et al., 2009; Zheng et al., 2025), potentially disrupting membrane fluidity/function and increasing cellular accumulation.

The dosing concentrations used in these studies are higher than measurements in human populations. Median human serum concentrations for PFAS can vary considerable dependent on population groups (0.1 ng/mL to 10,400 ng/mL) with many studies in the 1–100 ng/mL range (∼2.5–250 nM PFOA) (Jian et al., 2018; Piekarski et al., 2020). However, extremely high median concentrations of 1,000 to 10,000 ng/mL of PFOA (∼2.5–25 μM) have been found in the blood of occupational workers (Sakr et al., 2007; Zhou et al., 2014; Christensen and Calkins, 2023). While concentrations used in our experiments are not environmentally relevant to most human populations, these studies provide a novel model of PFOA neurotoxicity that could be extrapolated to chronic, low-dose models in future studies.

Additionally, removing the media supplement BIT9500, which contains an undisclosed amount of BSA, greatly lowered the EC50 value for PFOA by 10-fold but also reduced baseline viability by 50%. Several studies found that PFOA binds to serum albumin (Salvalaglio et al., 2010; Beesoon and Martin, 2015; Forsthuber et al., 2020) which can reduce PFAS cytotoxicity (Yang Y. D. et al., 2023). Although the removal of serum substitutes can increase the bioavailability of PFAS chemicals, it also adversely impacts normal viability and cell function. This poses a serious limitation on testing strategies to account for this binding of PFAS to serum proteins and estimation of PFOA entering the cell, making determination of the point of departure more difficult.

In addition to serum albumin (Salvalaglio et al., 2010; Beesoon and Martin, 2015; Forsthuber et al., 2020), PFAS can bind to fatty acid binding proteins (Sheng et al., 2016; Khazaee et al., 2021; Mann et al., 2022) and inhibit efflux transporters (Stevenson et al., 2006; Nakagawa et al., 2008; Yang et al., 2009; Weaver et al., 2010; Georgantzopoulou et al., 2014). It is possible that the increased uptake and retention of lipids is due to the interaction of PFOA with various transporters that results in their accumulation into the cells. These data highlight how serum proteins influence PFAS bioavailability and complicate *in vitro* toxicity assessments.

Exposure to PFOA increased lipid accumulation in phNPCs. This finding aligns with human epidemiological studies linking PFOA exposure to elevated cholesterol levels (Liu et al., 2023). Additional studies reported associations between PFAS levels and altered expression of genes involved in cholesterol transport (*NR1H2, NPC1, ABCG1, PPARA*) (Fletcher et al., 2013). Furthermore, PFOA and several other PFAS bind to nuclear receptors involved in lipid metabolism including PPARs (Wigger et al., 2019; Behr et al., 2020). Consequently, lipid accumulation is commonly seen after PFAS exposure in both animal and *in vitro* models, specifically in the liver (Attema et al., 2022; Wang et al., 2022; Sun et al., 2023; Yang W. et al., 2023). Although neurons have a low capacity to make LDs (Schönfeld and Reiser, 2013), Wu et al. (2024) found that PFOA increased LDs in hiPSC-derived neurons and altered glycerophospholipids and sphingolipids abundance (Wu et al., 2024).

Neuronal differentiation is a complex biological process involving transcriptional, epigenetic and metabolic reprogramming. As neuronal stem cells shift toward terminal neuronal differentiation, they undergo a shift from glycolysis to oxidative phosphorylation (Rajan and Fame, 2024).

During this period of neuronal differentiation, changes in mitochondrial form and function coincide with changes in metabolism with mitochondria forming more complicated branched and elongated structures as progression into neurons (Son and Han, 2018; Armat et al., 2025). Neurons produce high levels of reactive oxygen species, which causes the peroxidation of fatty acids which they are particularly suspectable to accumulate due to their limited ability to process lipids (Reynolds and Hastings, 1995). The transfer of this toxic lipid species from neurons to astrocytes can protect neurons from excess lipid peroxidation (Bailey et al., 2015; Ioannou et al., 2019).

PFAS exposure can lead to excitotoxicity and disruption of calcium homeostasis in different neuronal cell models (Harada et al., 2006; Liu et al., 2011; Dusza et al., 2018; Fang et al., 2018; Berntsen et al., 2020), suggesting further evidence of a role of ROS production. Excessive ROS can lead to disruptions of mitochondrial function and morphology including fragmentation of mitochondria indicative of mitophagy activation (Meng et al., 2025; Wen et al., 2025). We demonstrated that PFOA exposure can lead to an increase in mitochondrial ROS, lipid peroxidation, and more fragmented mitochondria. Souders et al. (2021) showed a decrease in mitochondrial membrane potential and an increase in ROS in neuronal SH-SY5Y at similar concentrations and timepoints used in our study (Souders et al., 2021). Similarly other studies have shown mitochondrial damage as a result of PFOA exposure in different model systems (Salimi et al., 2019; Zhao et al., 2022; Zhou et al., 2022; Rickard et al., 2025). PFOA exposure may induce lipid peroxidation, potentially involving mitochondrial reactive oxygen species (ROS) in the disruption of calcium homeostasis; however, the precise causal mechanisms—whether lipid accumulation, elevated mitochondrial ROS, impaired calcium regulation, or their interplay—remain unclear and require further investigation.

Inhibiting lipolysis or autophagy reduced lipid accumulation in PFOA-treated (but not vehicle- or OA-treated) phNPCs. This implies PFOA-stressed cells mobilize lipids *via* these pathways, possibly storing them in LDs—consistent with PFOA-induced autophagy from ER stress (Yan et al., 2017; Weng et al., 2020; Zhou et al., 2022; Qi et al., 2023; Qian et al., 2025), including a study in astrocytes where PFOA stimulated proinflammatory cytokine production (Min et al., 2025).

Using complex random mixtures of 47 high-risk environmental chemicals, our lab found that PFOA accumulated in the embryonic brain, altered embryonic brain weight and altered neuronal proliferation and differentiation (Hu et al., 2025). A goal of our current study was to elucidate the specific mechanism by which PFOA alters the balance of human progenitors and neurons. There are reported increases in the proliferation of PFOS-treated NPCs (Wan Ibrahim et al., 2013; Pierozan and Karlsson, 2021) but not PFOA-treated NPCs (Pierozan and Karlsson, 2021) and increases in proliferation and apoptosis in cerebral organoids treated with a PFAS mixture containing PFOA (Lu et al., 2024). We likewise found that PFOA exposure drastically decreased the number of neurons during differentiation and that neurons were more sensitive to PFOA than NPCs. This trend of decreased neuronal differentiation was also observed after PFOA exposure in SH-SY5Y neurons (Zhao et al., 2022), hiPSC-derived neurons (Wu et al., 2024) and cortical organoids exposed to a PFAS mixture (Lu et al., 2024).

It remains unclear how developmental PFOA exposure decreases neurogenesis but it is possible that proliferative NPCs can maintain a population of cells amid xenobiotic insult while post-mitotic neurons succumb to lipotoxicity or mitochondrial stress, given their high ROS and limited LD capacity (Reynolds and Hastings, 1995; Schönfeld and Reiser, 2013; Beckhauser et al., 2016). Utilizing phNPCs from different donor lines, we observed a negative correlation between BODIPY intensity and MAP2 gene and protein expression, suggesting a negative association between LD formation and neuron production after PFOA exposure. LDs are highly abundant in NPCs and decrease during neuronal differentiation. When LD production is increased, this can promote NPC survival and neuronal differentiation (Ramosaj et al., 2021). Conversely, lipid accumulation may also protect NPC and other glia cells against cell stress (Islimye et al., 2022). Therefore, PFOA lipotoxicity may present a challenge to NPCs in how to manage the need for lipid energy stores and generation of oxidative stress.

## 5 Conclusion

PFOA exposure in phNPCs reduced neuronal differentiation without affecting NPC proliferation, indicating specific vulnerability of neuron formation. This supports *in vivo* and other *in vitro* evidence of neuronal sensitivity to PFOA (Hu et al., 2025; Lu et al., 2024; Wu et al., 2024; Zhao et al., 2022). Aberrant lipid metabolism, including lipotoxicity likely contributes to decreased neurogenesis. A recent study using human cerebral organoids demonstrated that chronic exposure to a mixture of PFAS (PFOA, PFOS, and PFHxS) resulted in increased LDs, disrupted lipidomic profiles (particularly in sphingolipid metabolism), elevated amyloid-β (Aβ) accumulation, and increased phosphorylated tau (p-tau) levels, indicative of Alzheimer’s disease-like neuropathology (Lu et al., 2024). While our results suggest an effect between lipid metabolism and neuronal differentiation after PFOA exposure, *in vitro* models are inherently more limited in biological complexity then *in vivo* models, requiring follow-up work to examining direct effects on neurogenesis during critical windows of development. Nevertheless, *in vitro* models allow for future high-throughput studies with phNPC that are not possible with *in vivo* work. As with our previous work in phNPCs (Wolter et al., 2023 for lithium), future studies will examine the impact of donor genetic variability on phenotype, specifically LD formation and neuronal outcomes.

## Data Availability

The raw data supporting the conclusions of this article will be made available by the authors, without undue reservation.

## Supplementary material

Supplemental Figure 1 PFOA cytotoxicity is dependent on chain groups/length, multiple cell death pathways, and serum supplementation. (A) Human NPCs exposed to PFBA, PFBS, PFHxA, PFHxS, PFOA, or PFOS for 48 h and then assessed for viability and cytotoxicity using Alamar blue and LDH assays, respectively. PFAS chemicals with a longer chain or sulfonate head group are cytotoxic (lower EC50 values) than short chain or carboxylic compounds (n = 3 donors). (B) Co-Inhibition of necrosis, apoptosis, or necrosis improved viability in NPCs exposed to PFOA (n = 6 donors) for 48 h, increasing the EC50 values relative to controls in the Alamar blue assay. (C) Removing media supplementation (BIT9500) from the media greatly reduces cell viability lowering the EC50 when co-treating with PFOA (n = 3 donors). Nonlinear regression was performed with the Hill slope curve fitting for the EC50 value to generate dose-response curves. Error bars represent mean ± SEM. Each line represents the dose-response curve for each condition. All values are averages of replicates expressed relative to the viability of the untreated control cells.

Supplemental Figure 2 PFOA exposure increased lipid droplet intensity, area covered, and size in human NPCs. Human NPCs (n = 400–700 cells per condition from 19 donors) treated with 30–300 µM PFOA or vehicle (CTRL) resulted in an increase in (A) BODIPY intensity, (B) area covered by lipid droplets, or (C) size of lipid droplets. Human NPCs were pulsed with 300 µM PFOA then washed and chased for 24 or 48 h to determine the effect on lipid accumulation after chase. After removing PFOA, lipid droplet accumulation returned to baseline conditions, resulting in no significant differences in (D) BODIPY intensity, (E) percent of area covered by lipid droplets or (F) the size of the lipid droplets compared to controls (n = 70–100 cells per condition from 4 donors). Error bars represent mean ± SEM. Two-way ANOVA was conducted with sex and exposure as factors. Dunnett’s post hoc test compared to controls collapsed across sex: ****p < 0.0001, n.s = not significant.

Supplemental Figure 3 PFOA exposure decreased lipid turnover and resulted in more compact, fragmented mitochondria. (A–C) PFOA exposure (n = 50–80 cells per condition from 4 donors) for 48 h did not alter the lipid accumulation after the OA pulse however, once chased PFOA-exposed cells had an increase in **(A)** BODIPY intensity, **(B)** area covered by lipid droplets, **(C)** LD size compared to vehicle controls. Exposure to PFOA (30–1,000 µM) (n = 70–130 cells per condition from 6 donors) for 48 h resulted in a reduction in branch length **(D)**, increased compactness **(E)**, and decreased mitochondria footprint **(F)**, compared to vehicle controls (CTRL) but no differences in rotenone-treated cells (Rot). Error bars represent mean ± SEM. For pulse-chase experiment, a two-way ANOVA was conducted with exposure and timepoint as factors. Dunnett’s *post hoc* test compared to controls within timepoint: **p < 0.01, ***p < 0.001, ****p < 0.0001, n.s = not significant. For mitochondrial ROS experiment, one-way ANOVA was conducted and a Dunnett’s *post hoc* test compared to controls collapsed across sex: **p < 0.01, ***p < 0.001, ****p < 0.0001. A two-tailed t-test was conducted comparing CTRL to positive controls (CHP) or (Rot).

Supplemental Figure 4 The impact of lipid pathway inhibitors on PFOA-associated LDs. Treatment with lipid pathway inhibitors and PFOA (n = 100–200 cells per condition from 4 donors). (A–D) Exposure to FASN inhibitor cerulenin (FASNi) resulted in a reduction in (A) number of lipid droplets (LDs) in all exposure conditions but only decreased BODIPY intensity (B) and area covered by lipid droplets (C) in OA-treated NPCs and had not effect on LD size in any conditions (D). (E–H) Exposure to lipolysis inhibitor atglistatin (ATGLi) lead to an increase in number of LDs (E) and BODIPY intensity (F) in control- and OA-treated cells but an decrease in PFOA-treated NPCs. Treatment with ATGL also led to a decrease in LD area covered in PFOA-treated (G) cells and no effect on LD size in any conditions (H). (I-L) Exposure to autophagy inhibitor 3-methyladenine (ATGi) resulted in a reduction in number of LDs (I), BODIPY intensity (J) in only PFOA-treated cells. Treatment with 3 MA also resulted in an increased in LD area covered (K) in OA-treated cells and increase in LD size in vehicle-treated cells (L). (M-P) Fatty oxidation inhibitor etomoxir (CTPi) resulted in an increase in LDs in OA-treated cells (M), increases in BODIPY intensity (N) in control- and OA-treated cells, area covered (O) and LD size (P) in control-cells but no impact on PFOA-treated cells. (Q-T) Diglyceride acyltransferase-1 (DGAT1i) greatly reduced the (Q) number of LDs, (R) BODIPY intensity, (S) area covered and (T) LD Size in NPCs while DGAT2i slightly attenuated these parameters. Error bars represent mean ± SEM. Two-way ANOVA was conducted with exposure and inhibitor as factors. Dunnett’s *post hoc* test compared the difference between CTRL and exposure groups with and without the inhibitor: *p < 0.05, **p < 0.01, ***p < 0.001, ****p < 0.0001, n.s = not significant.

Supplemental Figure 5 Representative images. Co-exposure to vehicle (CTRL), 300 µM PFOA or 500 µM OA and co-treatment with lipid pathway inhibitors FASN inhibitor cerulenin, lipolysis inhibitor atglistatin (ATGLi), autophagy inhibitor 3-methyladenine (ATGi), fatty oxidation inhibitor etomoxir (CTPi), and Diglyceride acyltransferase-1/2 inhibitors (DGAT1/2i) for 48h, stained with BODIPY493 and DAPI.

Supplemental Figure 6 PFOA exposure had limited effects on NPC proliferation. (A) In human NPCs (n = 12 donors) there was no effect of PFOA exposure (10–300 µM) on the number of Ki67+ cells after 2, 4 or 6 days of exposure but there was a significant reduction in number of Ki67+ cells after 8 days of 300 µM PFOA exposure. **(B)** PFOA exposure had no effect of PFOA exposure on the number of EdU + cells after 2 or 4 days of exposure but there was a significant reduction in number of EdU + cells after 6 or 8 days of 300 µM PFOA exposure. **(C)** PFOA exposure had no effect of exposure on the number of pHH3+ cells after 2, 4, 6, or 8 days of PFOA exposure. **(D)** Representative image of NPCs exposed to 300 µM PFOA treated and vehicle-treated (CTRL) after 8 days of exposure and labelled with Ki67, EdU, pHH3 antibodies. **(E)** PFOA exposure (0.3–300 µM) for 4 days (n = 10 donors) did not result any changes to cell cycle using flow cytometry using EdU+, DyeCycle Violet + staining. **(F)** Representative dot plot of EdU vs. DyeCycle Violet for NPCs exposed to 300 µM PFOA and vehicle-treated (CTRL) after 4 days of exposure. **(G)** NPCs (n = 6 donors) were place in quiescence inducing media to examine how entry/exit into quiescence affected proliferation. **(H)** Treatment of human NPCs for 4 days with PFOA had number of Ki67+ cells in proliferation (NPC), quiescence exit (NPC entry), quiescence entry (qNPC entry), or quiescence (qNPC) media conditions. **(I)**. Treatment of human NPCs for 4 days with PFOA resulted in a decrease in pHH3+ cells in 300 µM PFOA treated NPC media conditions but no impact on NPC entry, qNPC entry, and qNPC media conditions. Error bars represent mean ± SEM. Dunnett’s *post hoc* test: *p < 0.05, **p < 0.01.For IHC experiments **(A–D)**, a two-way ANOVA was conducted with timepoint and PFOA exposure as factors, followed by a Dunnett’s *post hoc* test comparing exposure groups to control group at each timepoint: *p < 0.05, **p < 0.01. For flow cytometry experiment **(E–F)**, a two-way ANOVA was conducted with sex and PFOA exposure as factors, followed by a Dunnett’s *post hoc* test compared to controls collapsed across sex. For the quiescence experiments **(G–I)**, a two-way ANOVA was conducted with cell state (i.e., NPC entry) and PFOA exposure as factors, followed by a Dunnett’s *post hoc* test comparing exposure groups to control group within each cell state: **p < 0.01.

Supplemental Figure 7 PFOA exposure resulted in neurons with less branching morphology. Human NPCs were maintained in NPC media (NPC) or changed to neuronal differentiation media and treated with PFOA exposure or vehicle (CTRL) for 14 days (A) Cells treated with PFOA (0.3–300 μM, n = 16 donors) resulted in a reduction in proportion of MAP2+/SOX2+ cells with most reductions (B) in MAP2+ cells being SOX2-negative cells. PFOA (30–300 µM) also altered neuronal morphology by reducing the number of branching nodes (C) and mean branch length (D). Exposure to PFOA (10–300 µM PFOA, n = 17 donors) resulted in no changes in SOX2(E), PAX6(F), DCX(G), or NES (H) gene expression compared to vehicle-treated controls. Error bars represent mean ± SEM. Two-way ANOVA was conducted with sex and exposure as factors. Dunnett’s post hoc test compared to controls collapsed across sex: *p < 0.05, **p < 0.01, ***p < 0.001, ****p < 0.0001. A two-tailed t-test comparing CTRL to negative control using NPC Media controls (NPC): ^ααα^p<0.001, ^αααα^p<0.0001.
