# Supplementary figures and images for: Perfluorooctanoic acid (PFOA) induces lipid accumulation, oxidative stress, and reduced neurogenesis in primary human neuronal progenitor cells
Source: Front Toxicol. 2026 May 15;8:1814052. doi: 10.3389/ftox.2026.1814052 (PMC13218692; doi:10.3389/ftox.2026.1814052)

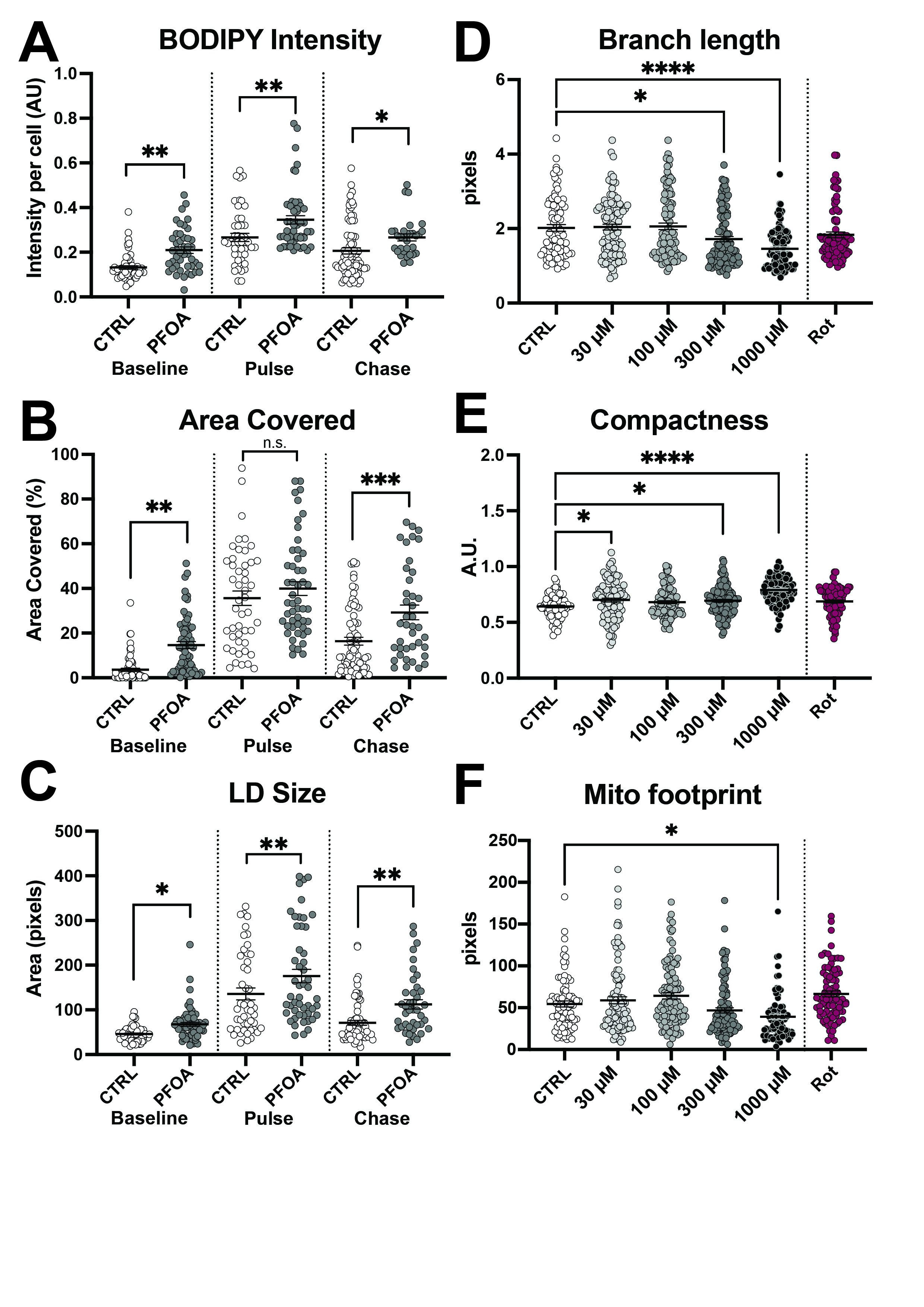

Supplement: Supplementary file 1 [file Image3.jpeg]

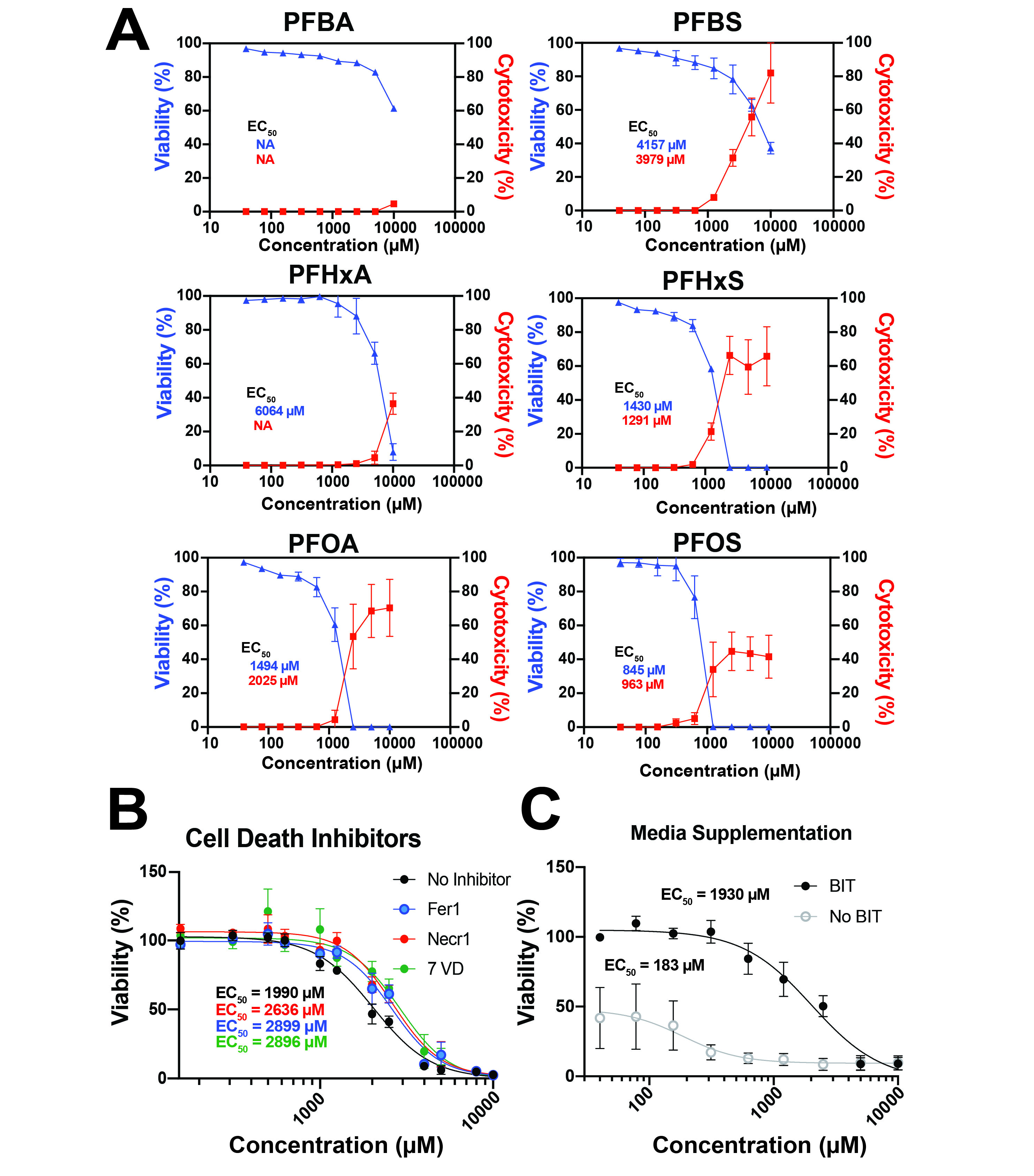

Supplement: Supplementary file 2 [file Image1.jpeg]

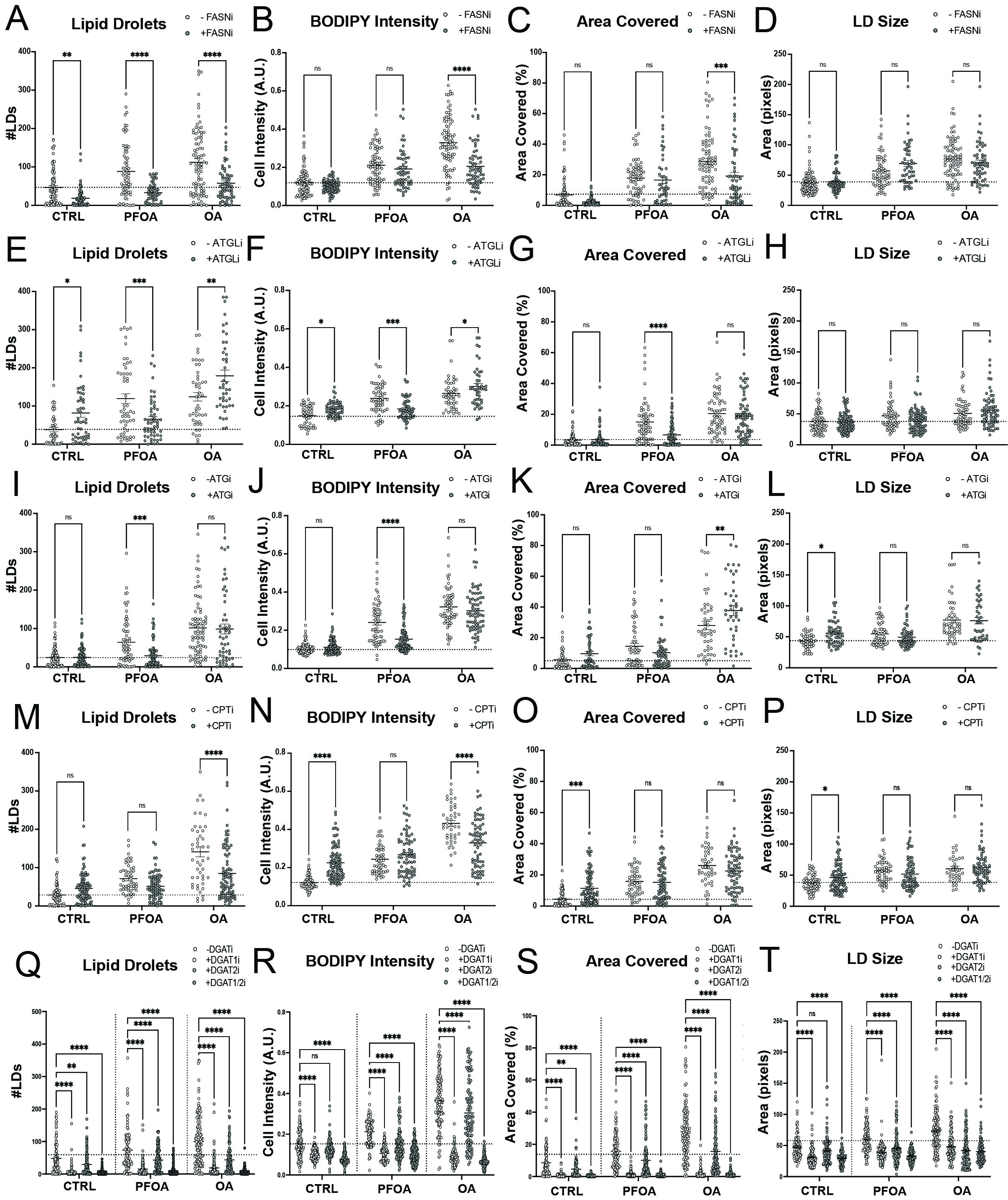

Supplement: Supplementary file 3 [file Image4.jpeg]

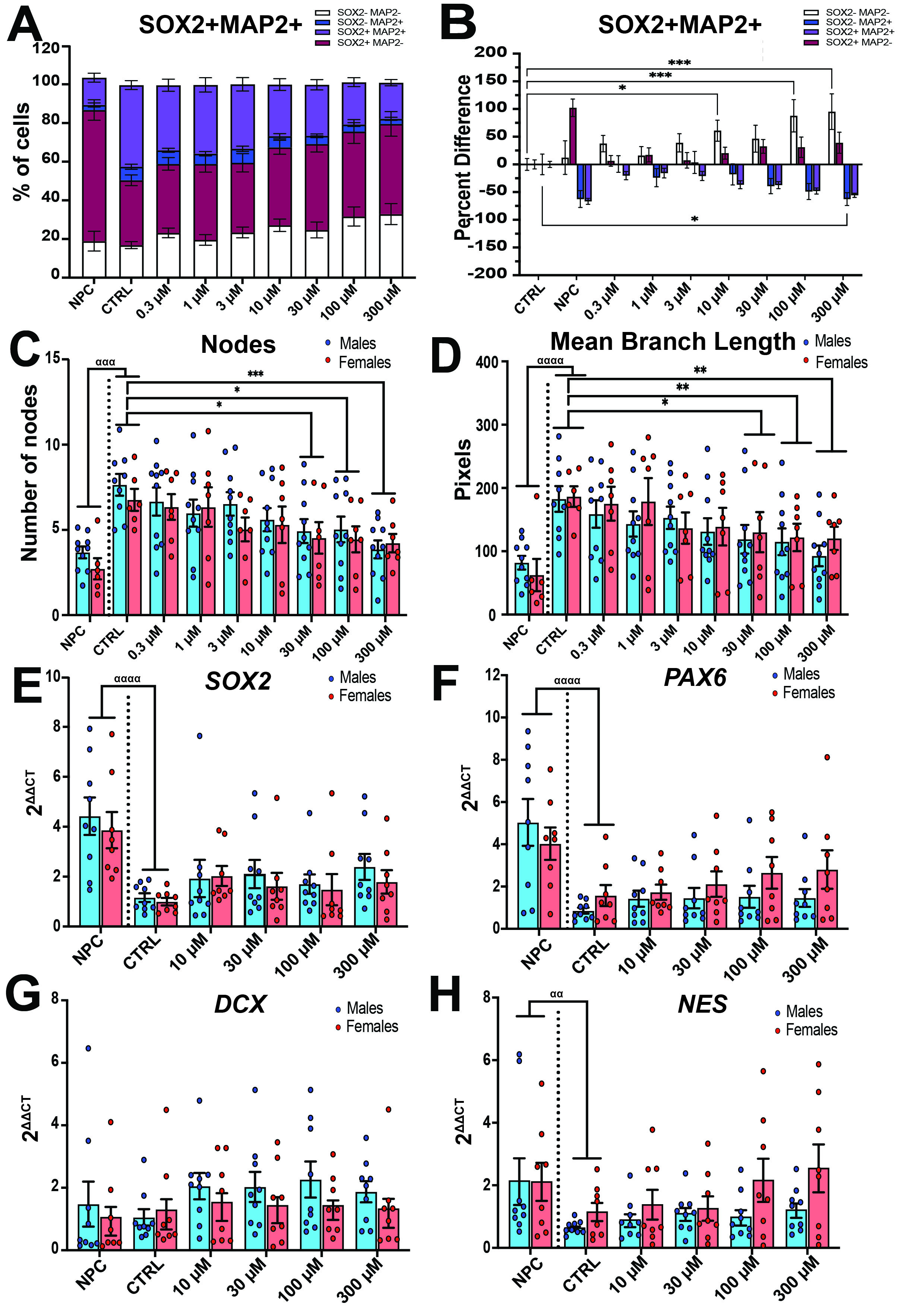

Supplement: Supplementary file 4 [file Image7.jpeg]

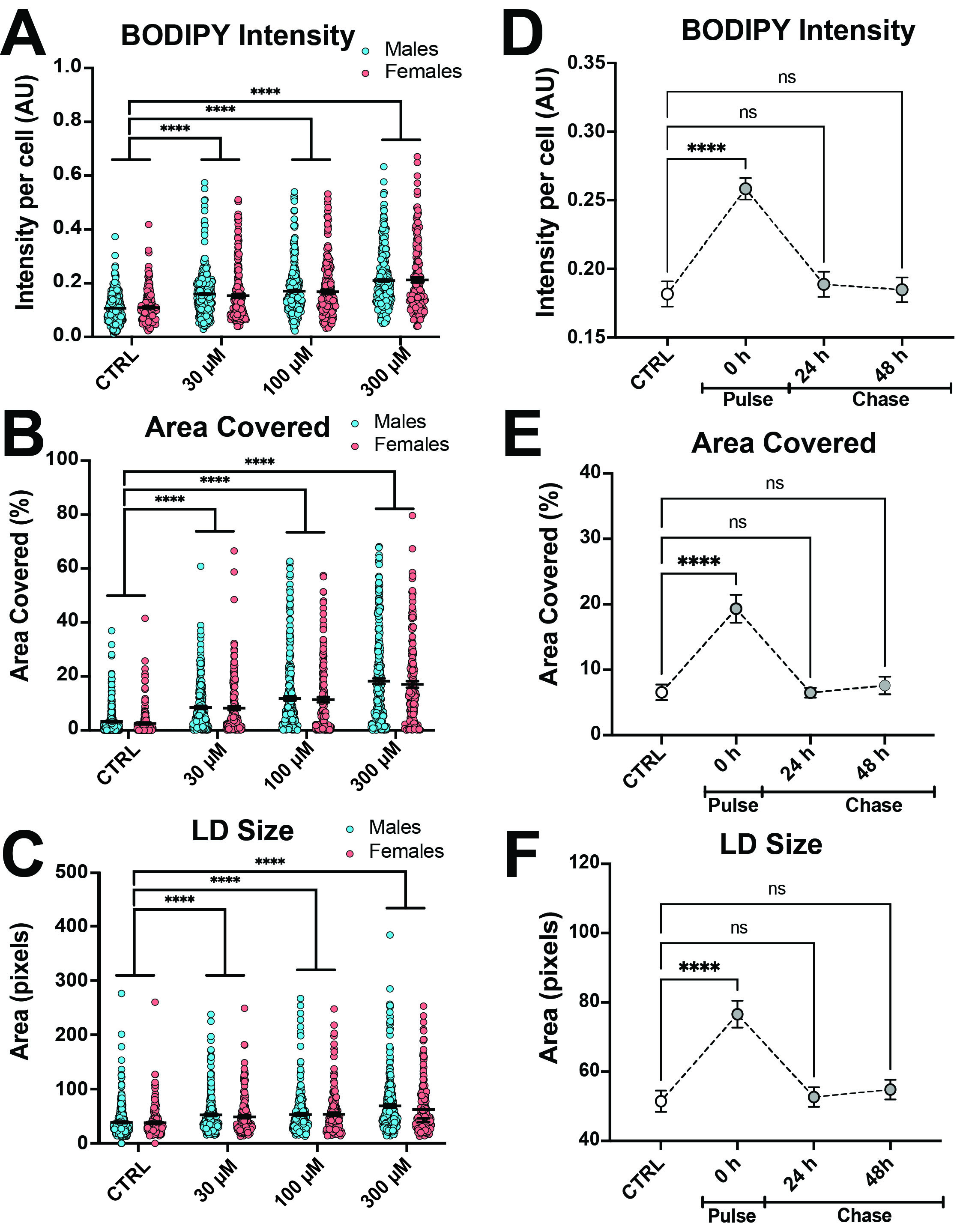

Supplement: Supplementary file 5 [file Image2.jpeg]

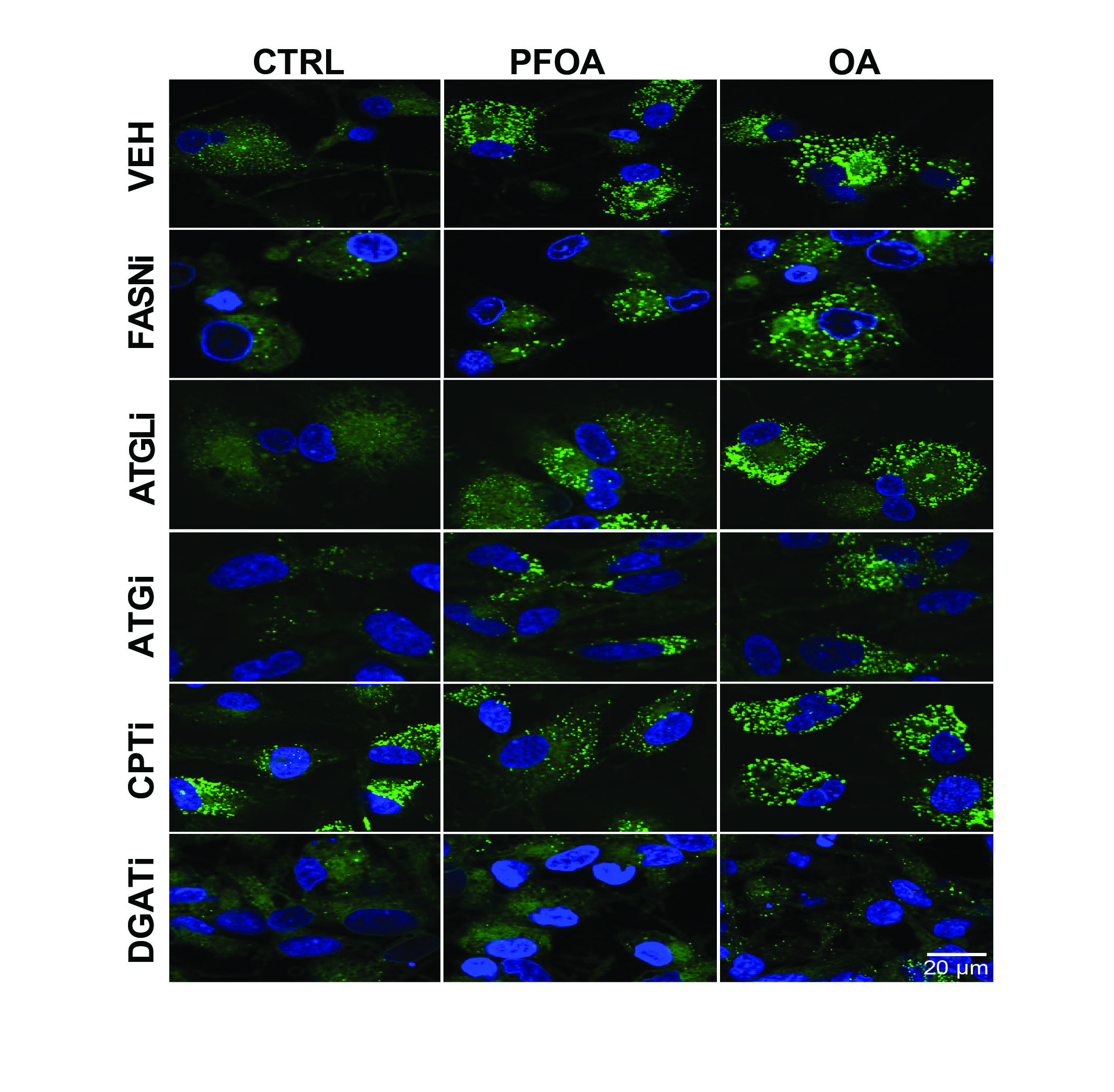

Supplement: Supplementary file 6 [file Image5.jpeg]

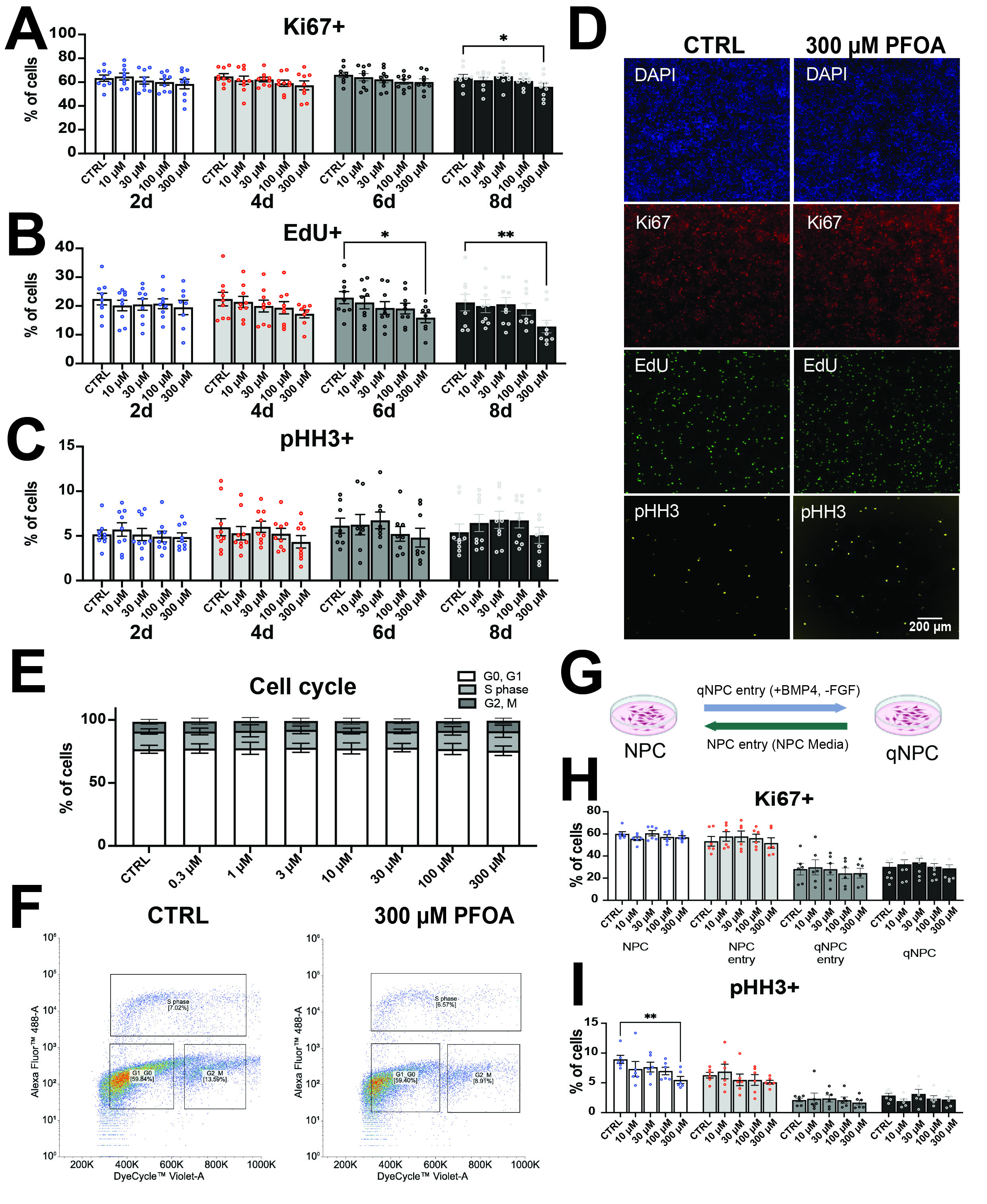

Supplement: Supplementary file 7 [file Image6.jpeg]
